# Integrating Heterogeneous Real-World Cancer Data for Semantic Interoperability in Oncology and Medical Imaging: Development and Validation of the Cancer Image Europe Hyperontology

**DOI:** 10.2196/85607

**Published:** 2026-09-08

**Authors:** Mirna El Ghosh, Varvara Kalokyri, Maciej Bobowicz, Melanie Sambres, Morgan Vaterkowski, Olga Giraldo, Jean Charlet, Laure Fournier, Catherine Duclos, Xavier Tannier, Gianna Tsakou, Manolis Tsiknakis, Ferdinand Dhombres, Christel Daniel

**Affiliations:** 1 Sorbonne Université, Université Sorbonne Paris-Nord, Inserm, Limics, 15 Rue de l'École de Médecine, Paris, 75006, France, 33 01 44 27 91 13; 2 Institute of Computer Science, Foundation for Research and Technology Hellas, Heraklion, Greece; 3 2nd Division of Radiology, Gdańsk Medical University, Gdansk, Poland; 4 Division of Radiooncology/Radiobiology, German Cancer Research Center, Heidelberg, Germany; 5 Direction de la Recherche et de l'Innovation, Assistance Publique—Hôpitaux de Paris, Paris, France; 6 Hôpital Européen Georges Pompidou, PARCC UMRS 970, Inserm, Université Paris Cité, Assistance Publique—Hôpitaux de Paris, Paris, France; 7 Université Sorbonne Paris-Nord, Assistance Publique—Hôpitaux de Paris, Avicenne, Santé Publique, Sorbonne Université, Inserm, Limics, Bobigny, France; 8 MAGGIOLI S.P.A.—Greek Branch, Research and Development Lab, Marousi, Greece; 9 Greater Paris Teaching Hospital (Assistance Publique—Hôpitaux de Paris), Medical Information Department, Hôpitaux Universitaires Henri-Mondor, Creteil, France

**Keywords:** Findable, Accessible, Interoperable, Reusable, artificial Intelligence, semantic interoperability, data heterogeneity, health care data standards, hyperontology, ontology-driven conceptual modeling, oncology, medical imaging, cancer imaging

## Abstract

**Background:**

Semantic interoperability in health care, essential for seamless integration of information systems, is partially achieved through the use of terminologies and common data standards that define the semantic structure of data. Various complexities arise when using real-world health care data, including different interpretations of terms and concepts and gaps in domain coverage in standard terminologies. However, ensuring compatibility becomes increasingly challenging when big data are distributed across diverse repositories that use heterogeneous health care standards and overlapping terminologies. Ontologies are key solutions to bridge these gaps, enabling consistent semantic interoperability and data harmonization.

**Objective:**

We aim to develop and validate a hyperontology within the EUCAIM (Cancer Image Europe) project to semantically integrate and harmonize clinical, biological, and imaging metadata, along with associated data from heterogeneous, disparate cancer image data models, to achieve semantic interoperability in oncology and medical imaging. The hyperontology will be used to support several EUCAIM components, including the extract, transform, and load process; federated query; image annotation and segmentation; and ultimately, AI-federated processing.

**Methods:**

The ontology development process combines real-world data from a network of European projects on cancer imaging (AI for Health Imaging) with their semantic mappings, as well as conceptual unpacking and modeling of the Minimal Common Oncology Data Elements (mCODE) specifications. The mCODE is a core set of structured data elements for oncology electronic health records. The building process is supported by ontology grounding, layering, and modularization. We adopted this hybrid approach to simplify ontology design, semantically reflect oncology’s essential entities and their interactions, and enhance the extensibility and reusability of the hyperontology. We initiated ontology development with a set of competency questions derived from the provided knowledge, which helped clarify the ontology’s scope and requirements and identify inconsistencies or incomplete information. We also assessed whether the requirements were fulfilled by formalizing the competency questions using SPARQL.

**Results:**

We developed a FAIR hyperontology that semantically integrates and harmonizes clinical, biological, and imaging metadata and data spread across disparate sources. The ontology also captures and accurately represents oncology and medical imaging. The hyperontology, which covers various cancer types, is rich in axiomatizations and patterns, supporting the semantic understanding and harmonization of heterogeneous data. Additionally, semantic mappings are established across data models and standards, ensuring the efficient and meaningful sharing and integration of health care data. Finally, we evaluated the ontology model and demonstrated its applicability using real-world prostate and breast cancer use cases.

**Conclusions:**

EUCAIM’s hyperontology is a valuable effort that provides a unifying framework for the essentials of oncology and medical imaging, facilitating communication among disparate and heterogeneous cancer image data models. The ontology model is evaluated and validated using multiple methods, demonstrating compliance with the specified ontological requirements. Challenges include ensuring that the ontology is scalable, extensible, and applicable, given the complexity and dynamic nature of the application domain.

## Introduction

With the rapidly increasing use of health care big data for artificial intelligence and predictive medicine, semantic interoperability is pivotal to ensuring seamless, accurate data integration and exchange across heterogeneous, disparate health care information systems. However, achieving compatibility becomes challenging in complex domains where heterogeneity affects data interpretation, standardization, and specification with different granularity levels. Semantic interoperability simplifies the coding, transmission, and use of meaning across seamless health services [1]. It enables information systems to share, understand, interpret, and use data unambiguously [2]. This is achieved by linking each piece of information to standardized terminology or vocabulary agreed at the international level (eg, Systematized Nomenclature of Medicine Clinical Terms [SNOMED-CT], Logical Observation Identifiers Names and Codes [LOINC], and *International Classification of Diseases, 10th Revision* [ICD-10]) and aligned with the Findable, Accessible, Interoperable, Reusable (FAIR) principles [3]. Interoperability, a central notion of FAIR, denotes “the ability of data or tools from non-cooperating resources to integrate or work together with minimal effort” [3]. As a high-level FAIR principle, interoperability is implemented in health care through common data standards, such as the Observational Medical Outcomes Partnership (OMOP) Common Data Model (CDM) [4], maintained by the Observational Health Data Sciences and Informatics program; and Fast Healthcare Interoperability Resources (FHIR), maintained by the Health Level Seven [5]. While OMOP CDM supports transforming health data to be stored in a standard relational database, FHIR standardizes both the structure and exchange of data across health care systems. Both OMOP and FHIR support, to a certain extent, semantic interoperability by providing standardized ways to represent and harmonize health care data. They permit structuring different health-related entities using a fixed set of key categories, called domains in OMOP and resources in FHIR. These entities reflect relevant but generic aspects of health care, such as patient, person, observation, condition, measurement, procedure, medication, and drug. FAIR-compliant biomedical terminologies (eg, SNOMED-CT, LOINC, RxNorm, and ICD-10) are used as coding systems to explicitly specify the meaning of possible values in value sets defined in standard data models (terminology binding). Therefore, both data standards and biomedical terminologies promote semantic interoperability and support data standardization performed throughout the extract, transform, and load (ETL) process [6].

Although OMOP and FHIR standardize and structure health data for their respective use contexts, semantic interoperability remains challenging, especially when integrating disparate, heterogeneous health care data repositories. Data in heterogeneous formats (eg, nonstandardized or incomplete) are difficult to incorporate into such models without prior standardization or transformation. The issue is amplified when data are integrated at different granularities [7]. For instance, OMOP provides limited support for representing the complete taxonomy in various terminologies, such as LOINC and OMOP Genomic. Genetic variants, such as TP53 (tumor protein p53) and BRCA2 (breast cancer gene 2; BRCA2 DNA repair associated), are not classified in OMOP Genomic and LOINC, respectively. Moreover, concept specifications are continuously evolving, which obstructs efficient compatibility among health care applications and challenges data analysis and exploration. In OMOP, for instance, the classification of concepts into domains (eg, condition, measurement, and observation) or classes (eg, clinical finding and disorder) can change between vocabulary versions. As an example, *Surgical margin involved by tumor* (SNOMED-CT, 370109009) was reassigned from the *Condition* to the *Measurement* domain, and *Malignant tumor of breast* (SNOMED-CT, 254837009) was reclassified from *Clinical Finding* to *Disorder*.

Despite the challenges posed by data heterogeneity and complexity to semantic interoperability, integrating heterogeneous data from disparate sources is essential for health care digitalization, making the resolution of this issue urgent [7]. Thus, to achieve semantic interoperability, a key requirement is that information systems understand the semantics (ie, meanings) of information [8]. This requires the use of ontologies, which are prominent semantic solutions for bridging the gap and supporting the explicit and accurate representation, access, and integration of heterogeneous data at different levels of detail [9-12]. Ontologies have been used to achieve semantic interoperability in health information systems [13,14], including semantic data interpretation and understanding as well as clinical decision support and reasoning [15]. However, representing complex diseases such as cancers is challenging due to the domain’s multimodal complexity, including diverse types of clinical, biological, and imaging metadata, as well as the rapid growth in the volume of cancer data generated by health care and cancer research [16]. Nevertheless, ontologies are suitable for representing cancers because they support domain complexity and make it understandable to humans, including clinicians and researchers, as well as machines [16]. Various ontologies have been developed in oncology and radiology [16,17], including the Operational Ontology for Oncology (O3) [18,19], the Radiation Oncology Ontology (ROO) [20], and the Radiation Oncology Structures (ROS) [21]. Additionally, for radiology, studies rely on the radiological lexicon (RadLex) [22], a controlled terminology developed by the Radiological Society of North America to support the description of medical imaging.

To address data heterogeneity and complexity, we aim to develop a hyperontology for oncology and medical imaging within the EUCAIM (Cancer Image Europe) [23] project. The hyperontology’s goal is to capture the complex structure of data from 4 European projects (ProCAncer-I, Incisive, ChAImeleon, and EuCanImage), forming the core of EUCAIM’s distributed cancer imaging data repository within the AI for Health Imaging (AI4HI) Network [24,25]. EUCAIM’s hyperontology helps aggregate heterogeneous and disparate cancer image data and integrate them into a common semantic metamodel. The term hyperontology is initially defined by Kutz et al [26] as a “modular heterogeneous ontology design methodology, i.e., the construction of ontologies that have parts, or modules, written in different formalisms, and which are interlinked in complex ways.” For EUCAIM, the hyperontology is the outcome of a complex method. It is defined as a FAIR-compliant, well-founded domain ontology that faithfully and explicitly models and represents domain concepts and relationships, reflecting the reality of oncology and medical imaging, while also supporting logical reasoning, inference, and validation. The hyperontology enables the complex semantic integration of different types of information (eg, clinical, biological, and imaging, as well as metadata and data) and mappings across various health care and imaging standards (eg, OMOP, FHIR, and Digital Imaging and Communications in Medicine [DICOM]) and terminologies (eg, SNOMED-CT, National Cancer Institute Thesaurus [NCIT], RadLex, ICD-10, and *International Classification of Diseases for Oncology, 3rd Edition* [ICD-O-3]). In the design, the hyperontology is divided into distinct modules and layers with varying granularity, interconnected through diverse semantic relations. A well-founded methodology was initiated in prior work [27] and extended in this study to facilitate the development of the ontology model, ensuring its reusability and extensibility and addressing the challenges posed by the complexity and evolving nature of cancer data. The hyperontology, grounded in the specifications of Minimal Common Oncology Data Elements (mCODE) [28,29] and implemented using the high-level conceptual modeling language OntoUML [30,31], is evaluated and validated using multiple methods. The ontology model is also extended to new cancer types emerged from new data holders. Finally, the hyperontology will be used across diverse EUCAIM components, supporting applications such as ETL, federated querying, processing, and image annotation and segmentation.

## Methods

### Data Sources

We used 16 dataset collections from AI4HI [24,25] to design the hyperontology and validate our methodology. These collections cover cancer of the breast, prostate, lung, liver, colon, colorectal, and rectum. We collected different types of clinical, biological, and imaging metadata, such as cancer types and subtypes, histological subtypes, body parts, family history, comorbidities, specimen, tumor marker tests and results, genetic tests, cancer staging and grading methods and values, treatment, surgical and imaging procedures, and drugs. However, there is no standard for the amount and granularity of required data among the projects [24]. For instance, histology or morphology, medication, and follow-up are not commonly provided. Besides, in AI4HI, various data models and standards have been used, enabling the coexistence of different terminologies and vocabularies. While ProCAncer-I and ChAImeleon have adopted OMOP to harmonize and standardize their data, FHIR has been used in Incisive and EuCanImage. Despite standardization efforts, data heterogeneity persists, underscoring the lack of uniform health care data standards—a persistent issue that hinders interoperability. It also highlights the data disparity and complexity across independent, geographically dispersed sources, posing additional challenges for efficient interoperability. In Table 1, we present examples of data heterogeneity and complexity observed across projects adopting different data standards (eg, OMOP or FHIR) and within projects conforming to the same CDM or standard (eg, OMOP).

**Table 1.** Examples of heterogeneous and complex clinical or biological data provided by AI4HI^a^.

Category and variable	Domain or resource (standard)	Value	Standard source of variable or value (as provided by AI4HI)	AI4HI source
Results of tumor marker tests				
ER^b^ positive	Condition (OMOP^c^)	—^d^	SNOMEDCT:416053008^e^	ChAImeleon
ER	Observation (FHIR^f^)	Positive	LOINC:85337‐4^g^/SNOMEDCT:10828004	EuCanImage
Cancer staging methods				
AJCC/UICC^h^ 7th pathological M1a Category	Measurement (OMOP)	—	Cancer Modifier:p-7th_AJCC/UICC-M1a	ProCAncer-I
TNM Path M	Measurement (OMOP)	pM1a	NAACCR:900/NAACCR:900@p1A^i^	ChAImeleon
Genetic tests				
BRCA1^j^ (BRCA1 DNA repair associated) gene variant	Measurement (OMOP)	Present	OMOPGenomic:1100/SNOMEDCT:52101004	ChAImeleon
BRCA1 gene mutations tested for in Blood or Tissue by Molecular genetics method Nominal	Observation (FHIR)	Positive	LOINC:21639‐0/LOINC:LA6576-8	Incisive
BRCA1 mutation carrier detection test	Diagnostic Report (FHIR)	BRCA1 gene mutation positive	SNOMEDCT:405823003/SNOMEDCT:412734009	EuCanImage

a AI4HI: AI for Health Imaging.

b ER: estrogen receptor.

c OMOP: Observational Medical Outcomes Partnership.

d Not available.

e SNOMEDCT: Systematized Nomenclature of Medicine Clinical Terms.

f FHIR: Fast Healthcare Interoperability Resources.

g LOINC: Logical Observation Identifiers Names and Codes.

h AJCC/UICC: American Joint Committee on Cancer/Union for International Cancer Control.

i NAACCR: North American Association of Central Cancer Registries.

j BRCA1: breast cancer gene 1.

**Table 2.** Examples of ambiguous or nonstandardized clinical data provided by AI4HI^a^.

Category	Terms	AI4HI source
Breast cancer molecular subtypes	Luminal A, Luminal B (HER2^b^ negative or HER2 positive), HER2 positive (enriched)/HR^c^ negative (non-luminal), Triple negative	EuCanImage
Breast cancer triple-negative subtypes	Basal-like 1, Basal-like 2, Immunomodulatory, Mesenchymal-like, Mesenchymal stem-like, Luminal androgen receptor	ChAImeleon

a AI4HI: AI for Health Imaging.

b HER2: human epidermal growth factor receptor 2.

c HR: hormone receptor.

In addition to heterogeneity, ambiguous or nonstandardized data are captured, such as breast cancer subtypes (see Table 2 for examples).

Unlike other subtypes, such as *HER2-negative* (SNOMED-CT, 431396003) and *HER2-positive* (SNOMED-CT, 427685000), these terms lack formal definitions. However, clarifying their meaning is required to support accurate semantic specification and reduce ambiguities.

For imaging, we collected annotation and segmentation metadata, along with the corresponding values. This metadata is defined in DICOM (Digital Imaging and Communications in Medicine) [32], a standard for medical imaging that defines how images and associated information are stored and transmitted. Examples of imaging metadata are *Modality* (0008,0060), *Laterality* (0020,0060), and *Segment label* (0062,0005). Most DICOM metadata (eg, *Segment label* (0062,0005), *Segmentation type* (0062,0001), *Segmentation algorithm type* (0062,0008), and *Image type* (0008,0008)) is not formally specified in standard terminologies for imaging, such as RadLex [22], Semantic DICOM Ontology (SEDI) [33], and DICOM controlled terminology [34]. Recent efforts [35,36] are underway to extend OMOP for radiology, thereby filling the gap and enabling the definition and reuse of standardized imaging knowledge. We also collected imaging values or labels, such as “MR” (magnetic resonance) and “CT” (computed tomography) associated with Modality (0008,0060), and “Automatic,” “Semi-Automatic,” and “Manual” associated with *Segmentation algorithm type* (0062,0008). These values or labels can be mapped to standards, such as SNOMED-CT and RadLex.

### Methodology

#### Overview

We propose a methodology primarily inspired by Neon [37] and Systematic Approach for Building Ontologies (SABiO) [10] methodologies. While the former supports the reuse and restructure of ontological sources, knowledge integration, and collaboration in building evolving ontology networks, the latter focuses on a step-by-step process, ontological analysis, requirement traceability, and validation for developing domain ontologies. Both methods commonly initiate the development process with requirements specification and the use of competency questions (CQs) for functional requirements. Given the heterogeneity, complexity, and evolving nature of the application domain, we combined these methods into a single iterative hybrid methodology. The novelty lies in combining SABiO’s structured development, focused on ontological analysis and validation, with Neon’s dynamic scenario-based process, focused on reuse, mapping, and evolution. This combination creates a step-by-step, detailed methodology that is requirement-driven yet systematically and formally well-founded, thereby simplifying hyperontology development while maintaining semantic interoperability. Our methods integrate the metadata collected from heterogeneous data models and their associated semantic mappings with conceptual unpacking [38,39] and oncology modeling based on mCODE specifications [28,29]. Support processes, including ontology layering and modularization, have also been used to streamline the hyperontology development. Additionally, we have held regular meetings and discussions with EUCAIM partners and medical experts throughout the development and actively incorporated their feedback into the framework.

#### Ontology Requirement Analysis and Specifications

We initiated the ontology development by analyzing the ontological requirements and specifications (including purpose, scope, users, and uses). The functional requirements that specify the knowledge the hyperontology should represent are stated as CQs, classified by cancer type. We used a bottom-up approach to syntactically analyze the AI4HI collected data and formulate 285 knowledge-based, specific CQs and answers. Furthermore, these questions are generalized into 28 generic CQs (Textbox 1). Identifying CQs was an effective way not only for ontology scoping—that is, identifying ontologically relevant content—but also for capturing interactions, detecting inconsistencies and information gaps, including nonstandardized data, and evaluating the ontology. Additionally, nonfunctional requirements that address generic qualities and capabilities, such as FAIR compliance and language support, have been considered. Regarding the specifications, the hyperontology seeks to integrate and harmonize heterogeneous data by providing an unambiguous and faithful representation of oncology and medical imaging. It covers AI4HI and new datasets emerged from new data holders joining the EUCAIM community. Finally, the hyperontology will support the exploration of data collections, image annotation and segmentation, as well as federated querying and processing performed by data users or researchers. The Ontology Requirements Specification Document (ORSD) [40], initially proposed in Neon [37], illustrates all these requirements and specifications.

Textbox 1. **Generic competency questions (CQs) formulated in a bottom-up approach based on specific CQs illustrated in the Ontology Requirements Specification Document.**

**Cancer diagnosis**

- CQ1. What demographic details (age, sex, and gender) are collected to identify a patient with cancer?
- CQ2. What are the main types and subtypes of cancer conditions identified during the cancer diagnosis?
- CQ3. Are there any histology or morphology parameters or descriptors associated with cancer conditions?
- CQ4. Have any metastatic cancers been identified during the diagnosis?
- CQ5. What are the main body sites affected by cancer?
- CQ6. Have any comorbidities been identified during the diagnostic process?
- CQ7. Which laboratory tests are used to diagnose cancer?
- CQ8. Which tumor marker tests are used to diagnose patients with cancer?
- CQ9. Which gene variant measurements are used for cancer diagnosis?
- CQ10. What are the primary staging methods and associated values used for cancer classification?
- CQ11. What are the primary grading methods and associated values used for cancer classification?
- CQ12. Which information is gathered regarding family history?
- CQ13. How is the health performance status identified and classified?

**Imaging process**

- CQ14. Which diagnostic imaging procedures are used for cancer detection?
- CQ15. Which imaging tests are used for cancer diagnosis?
- CQ16. Which imaging results are associated with the imaging tests?
- CQ17. Which imaging assessment methods are used in cancer diagnosis?

**Imaging study**

- CQ18. What are the primary imaging modalities or techniques used for cancer image study?
- CQ19. What manufacturer is involved in the cancer imaging study?
- CQ20. How is the laterality specified?
- CQ21. Which patient positions are considered for image study?

**Cancer treatment**

- CQ22. Which surgical procedures are used for cancer treatment?
- CQ23. Which medication or drug therapy is used for cancer treatment?
- CQ24. Are there any standard dosing units for medication or drug therapy?
- CQ25. Which radiotherapy therapies are used for cancer treatment?

**After treatment**

- CQ26. What response to treatment has been identified?
- CQ27. How has the tumor progression been identified?
- CQ28. Has the date of the last contact been provided?

#### Knowledge Acquisition

**Figure 1.**
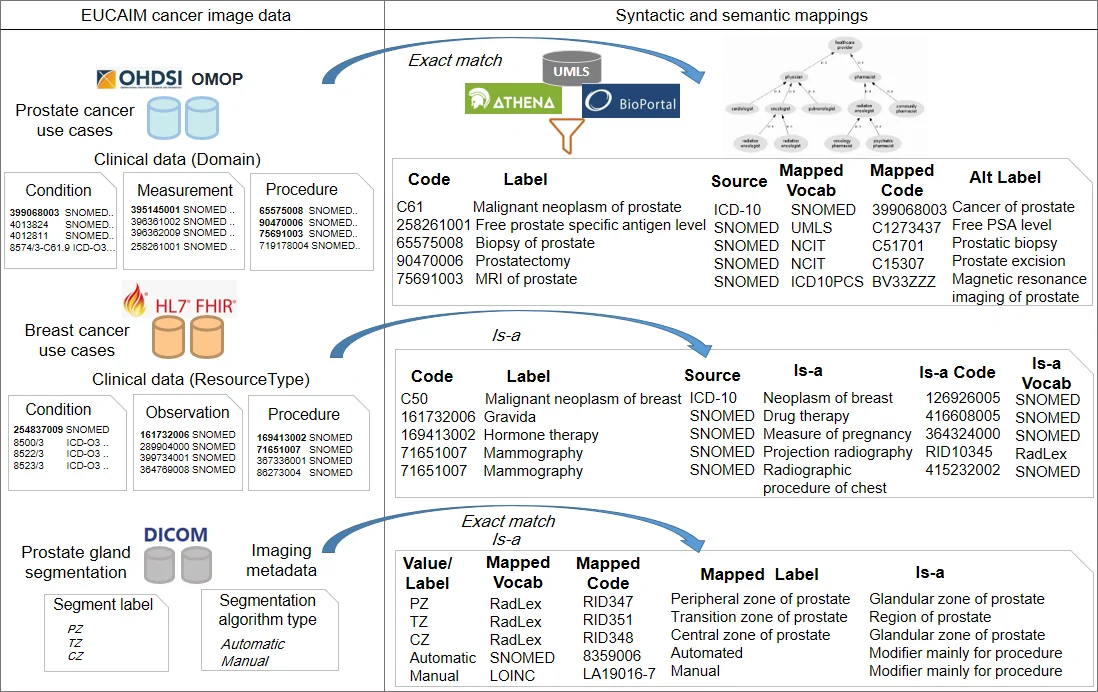
An illustration of mapping clinical and imaging data or metadata with standard sources. DICOM: Digital Imaging and Communications in Medicine; EUCAIM: Cancer Image Europe; FHIR: Fast Healthcare Interoperability Resources; HL7: Health Level Seven; ICD-10: International Classification of Diseases, 10th Revision; ICD-O-3: International Classification of Diseases for Oncology, 3rd Edition; ICD10PCS: International Classification of Diseases, 10th Revision, Procedure Coding System; LOINC: Logical Observation Identifiers Names and Codes; MRI: magnetic resonance imaging; NCIT: National Cancer Institute Thesaurus; OHDSI: Observational Health Data Sciences and Informatics; OMOP: Observational Medical Outcomes Partnership; PSA: prostate-specific antigen; RadLex: radiological lexicon; SNOMED: Systematized Nomenclature of Medicine; UMLS: Unified Medical Language System.

After specifying the ontological requirements, we began harvesting the knowledge needed to construct the hyperontology’s content and structure, primarily through mappings to standard biomedical terminological and ontological resources (eg, SNOMED-CT, LOINC, ICD-10, ICD-O-3, RadLex, and NCIT). We revised the collected knowledge in the ORSD to verify any incomplete information, such as missing labels, vocabulary, or code, and to prevent information loss. Furthermore, 2 main types of mappings have been applied: hierarchical (is-a), which focuses on harvesting taxonomic relations, and exact match mappings, which concentrate on alternative labels, codes, and definitions (Figure 1).

Additionally, considering the interactions captured from CQs, we identified semantic relationships across categories, accounting for their necessity and consistency to prevent the ontology from becoming excessively complex or extensive. For instance, “*Has associated morphology*” is an object property defined in SNOMED-CT and helps connect cancer conditions (eg, *Malignant tumor of breast* (SNOMED-CT, 254837009)) with their related histology and morphology (eg, *Malignant Neoplasm* (SNOMED-CT, 367651003)). Tumor morphology, which AI4HI did not commonly provide, is also collected from ICD-O-3 and SNOMED-CT to enrich and maintain this content, given its querying requirements and importance in supporting cancer diagnosis. For imaging, we mapped DICOM metadata to RadLex, a primary radiology standard recommended by experts. We also aligned the imaging values (labels) in the DICOM metadata with various standards (eg, SNOMED-CT and RadLex) using an exact-match similarity approach.

The mappings are performed automatically across multiple resources that combine many health and biomedical vocabularies and standards.:

- Observational Health Data Sciences and Informatics ATHENA [41] permits harvesting diverse mappings (exact search and is-a) from various standardized vocabularies.
- BioPortal RESTful API [42] comprises different resources (ontologies and classes) and related end points for accessing and browsing biomedical content. BioPortal helps enrich the hyperontology with synonyms, codes, and definitions and access the resources not considered in Unified Medical Language System (UMLS) or ATHENA (eg, RadLex and NCIT).
- UMLS REST API [43] provides end points for searching and retrieving UMLS content (eg, atoms, concept unique identifiers, definitions, semantic types, parents, and children).

Finally, we revised the results to detect differences or inconsistencies and manually curated them to remove any information irrelevant to the application domain. The curation process was challenging due to the complexity and heterogeneity in how concepts are represented and specified across standard terminologies. For instance, histology or morphology is interpreted differently in SNOMED-CT and NCIT. While in SNOMED-CT, morphologies are specified as body structures, NCIT classifies them as diseases or disorders, reflecting a semantic divergence and ambiguity [44]. The task was further complicated by the insufficiency or absence of hierarchical (“is-a”) classification in several sources, such as OMOP Genomic, North American Association of Central Cancer Registries, Cancer Modifier, LOINC, and Unified Code for Units of Measure. These sources are commonly used to standardize entities such as laboratory or genetic texts, histological or pathological stages or grades, topography, and measurement units.

The heterogeneity and complexity revealed among the obtained mappings have necessitated the involvement of medical experts knowledgeable about biomedical ontologies to supervise the curation process. However, some conflicting representations and classifications remained unresolved because the exact meanings and specifications of concepts needed to be clarified and explicitly described in relation to foundational and core specifications. This can be achieved through high-level conceptual modeling practices.

#### Design and Conceptualization

**Figure 2.**
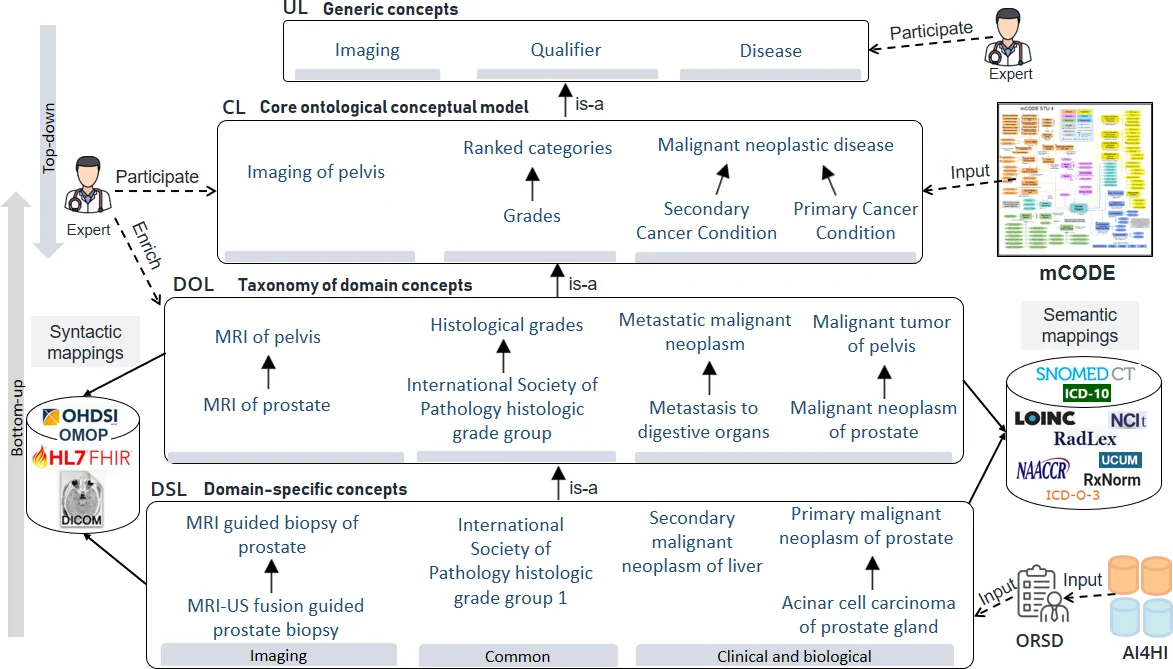
An illustration of the hyperontology design approach. AI4HI: AI for Health Imaging; CL: core layer; DICOM: Digital Imaging and Communications in Medicine; DOL: domain layer; DSL: domain-specific layer; FHIR: Fast Healthcare Interoperability Resources; HL7: Health Level Seven; ICD-10: International Classification of Diseases, 10th Revision; ICD-O-3: International Classification of Diseases for Oncology, 3rd Edition; LOINC: Logical Observation Identifiers Names and Codes; mCODE: Minimal Common Oncology Data Elements; MRI: magnetic resonance imaging; MRI-US: magnetic resonance imaging–ultrasound; NAACCR: North American Association of Central Cancer Registries; NCIT: National Cancer Institute Thesaurus; OHDSI: Observational Health Data Sciences and Informatics; OMOP: Observational Medical Outcomes Partnership; ORSD: Ontology Requirements Specification Document; RadLex: radiological lexicon; SNOMEDCT: Systematized Nomenclature of Medicine Clinical Terms; UCUM: Unified Code for Units of Measure; UL: upper layer.

The diversity and complexity of the application domain necessitated a design approach that enables a consistent yet simplified ontology-building process. In this regard, we applied ontology modularization [45], suggested in Neon [37] and SABiO [10], to facilitate the development process and support the ontology’s extensibility and reusability. However, we adopted a contextualized approach to organize the ontology into generic modular components (Figure 2), with a semantic separation among domain knowledge, shared cross-domain entities, and integration-oriented modeling constructs. Domain-specific biomedical knowledge is captured in the clinical and biological and imaging modules, while the common module provides cross-domain entities and shared value sets (eg, demographic attributes, measurement units, and qualitative result values) reused across modules. These modules, created by conceptual thematic grouping, are domain-based ontology parts designed to accommodate the various types of metadata provided by AI4HI. They establish explicit boundaries among areas of knowledge, with each module focusing on a specific set of domain-related concepts.

The clinical and biological module encompasses aspects such as cancer types and subtypes, histology and morphology, tumor marker tests and results, treatment and medications, family history, surgical and therapeutic procedures, and staging and grading methods. The *Imaging* module covers the essentials of the imaging domain, focusing on cancer, such as the imaging modalities (eg, *MR* (Magnetic resonance), *PET* (Positron emission tomography), and *CT* (Computed tomography)), as well as procedures, assessments (eg, *PI-RADS* (Prostate Imaging Reporting and Data System), *BI-RADS* (Breast Imaging Reporting and Data System), and *LI-RADS* (Liver Imaging Reporting and Data System)), and observations. Finally, the *Common* module defines patient demographics (eg, age, sex, gender, and race) and a wide range of qualifiers, such as staging or grading values (eg, *pM1a, pM1b*, *G1, G2, G3*, *1, 2*, and *3*), absence or presence findings (eg, *not assessed*, *not mutated*, and *present*), unit of measure (eg, *percent* and *milliliter*), as well as laboratory test results (eg, *positive*, *negative*, *1+*, and *2+*). Numeric classes (eg, 1, 2, 3, 1+, and 2+) included in this module correspond to value sets required by clinical staging systems and laboratory test results. These values, represented in the ontology, originate from datasets produced by the AI4HI projects (see ORSD version 1.1 [40]). In addition, 2 auxiliary modules (generic and specific) were introduced to support semantic integration with EUCAIM infrastructure and external data models by representing general observational structures, temporal events, and dataset-level metadata.

Hierarchically, we divide the ontology structure into 4 layers (Figure 2), each with varying granularity, ranging from the most specific to the most generic: domain-specific, domain, core, and upper. We adopted these layers based on Guarino’s classification of ontologies, regarding their level of independence for a particular task or point of view [9]. The domain-specific layer (DSL) determines the granularity level of the hyperontology. The cancer-related knowledge (clinical, biological, and imaging) provided by AI4HI is enclosed in this layer. The domain layer is built from the DSL’s content, considering the hierarchical (is-a) mappings harvested during the knowledge acquisition phase. We developed DSL and domain layer using a bottom-up strategy. We also enriched them with the resulting syntactic mappings using the Simple Knowledge Organization System (SKOS) profile [46], which permits annotating concepts with their preferred labels (*skos:prefLabel*), alternative labels or synonyms (*skos:altLabel*), and definitions (*skos:definition*). The oncology core concepts and relationships are represented in the core layer through the application of the *ontology unpacking process* [38]. This involves an ontological analysis that reveals the oncology ontological conceptual model, thereby ensuring semantic interoperability in accordance with FAIR principles [3]. OntoUML [30,31], a high-level ontology-driven well-founded conceptual modeling language, is used to ontologically analyze and represent the essentials of oncology, based on the mCODE specifications [28,29]. The OntoUML metamodel aligns with the Unified Foundational Ontology (UFO) [39], in which the OntoUML modeling primitives (eg, *category*, *kind*, *subkind*, *role*, *phase*, *mode*, *quality*, *event*, and *situation*) correspond to the ontological distinctions and axiomatization of UFO. UFO, which makes a fundamental distinction between endurants (objects or tropes) and perdurants (events), is a foundational ontology grounded in philosophical theories and developed to support conceptual modeling in various domains, including business process and ontology-driven modeling [30,31]. Based on FHIR, widely adopted by health care establishments that are increasingly implementing FHIR standards [47], mCODE is defined as a “Domain of Knowledge” implementation guide intended to demonstrate how to represent clinical concepts in general, with the aim of increasing interoperability in oncology.

The ontological analysis and conceptual modeling of oncology’s essential entities and their interactions are performed using a top-down approach, helping uncover the domain basics and resolving ambiguity. Finally, with expert help, the generic categories are maintained in the upper layer, the top level of the hierarchy. Combining bottom-up and top-down in a middle-out fashion has effectively supported a coherent ontological model that links oncology’s conceptual analysis with specific application knowledge.

#### Evaluation and Validation

Following SABiO [10], ontology evaluation verifies that the ontology is correctly built to meet the initial specifications, and validation ensures that it fulfills its intended purpose. While the former focuses on the internal quality of the ontology model, such as clarity, competency, and consistency, the latter ensures external validation of reusability and applicability, including its ability to represent real-world situations. We thereby incorporated multiple evaluation methods into a multidimensional strategy, including ORSD verification, ontology revision with experts, the formalization of CQs using the SPARQL Protocol and Resource Description Framework Query Language (SPARQL) [48], and consistency checking using logic reasoners. We also validated the ontology’s applicability to real-world cancer scenarios from AI4HI and its extendibility using new datasets.

### Ethical Considerations

This study used previously collected datasets that were anonymized before access. Datasets did not include personally identifiable information. The study adheres to data protection requirements and regulations, as well as ethical research standards.

## Results

### Core Ontological Conceptual Model

**Figure 3.**
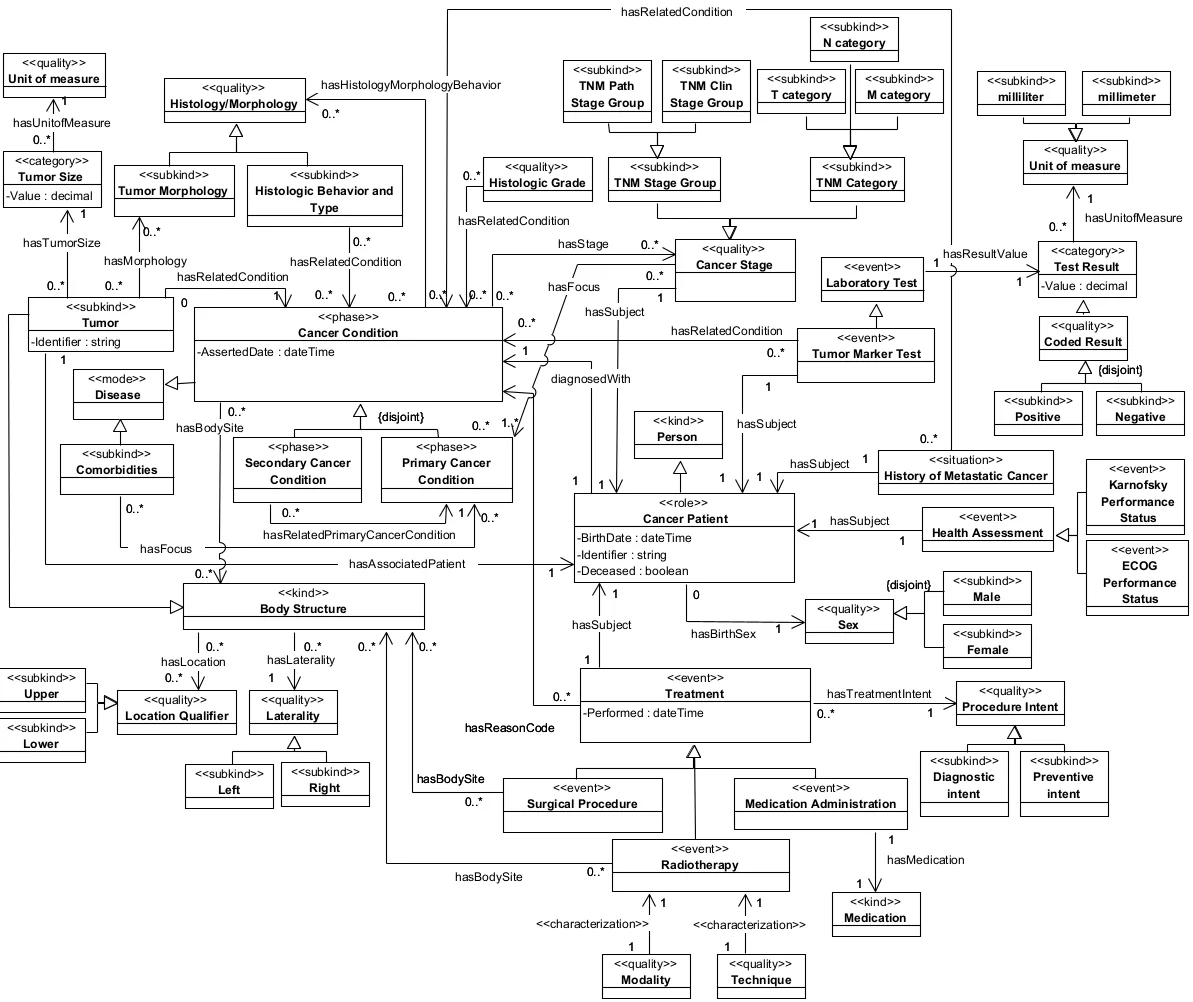
An illustration of part of the ontological conceptual model of the mCODE clinical aspects using OntoUML. ECOG: Eastern Cooperative Oncology Group; mCODE: Minimal Common Oncology Data Elements. *The maximum number of occurrences, meaning many or unbounded.

This section introduces clinical and imaging ontological conceptual models that serve as the ground for the hyperontology model in oncology and medical imaging. These models are developed in alignment with the mCODE specifications (Standard for Trial Use, Release 4) [29] and the structural content of DICOM imaging studies [49]. Figure 3 illustrates an OntoUML class diagram that captures key cancer-related concepts, addressing the generic CQs of the hyperontology concerning the following clinical aspects: (1) patient information, (2) disease characterization, (3) health assessment, (4) cancer treatments, and (5) outcomes. These aspects (detailed in previous work [44]) correspond to the thematic groups introduced in mCODE, which are provided as a set of templates or patterns, including controlled terminologies, reflecting the basics of oncology and are extendable to cover additional or specific aspects.

Patient Information defines basic details about the patient, such as the *identifier*, demographics (eg, *birth date*, sex at birth, *gender*, *race*, and *ethnicity*), and the vital status. In mCODE, a *Patient* has been diagnosed with or is receiving medical treatment for a malignant growth or tumor.

Disease Characterization defines data elements specific to the diagnosis, characteristics, and staging of cancer. The diagnosis includes *Primary Cancer Condition* (the original neoplasm in the patient’s body) and *Secondary Cancer Condition* (resulting from the spread [metastasization] of cancer from its original site). The latter depends on the former, being the main related cancer condition. Cancer conditions located at a specific body site, characterized by location and laterality qualifiers*,* have morphologic and behavioral characteristics (*Histologic Behavior and Type, Tumor Morphology,* and *Histologic Grade*). They are further characterized by *Cancer Stage,* including *TNM Category* and *TNM Stage Group. The Tumor Marker Test*, performed for cancer conditions, yields either a coded test result (eg, positive and negative) or a quantitative result.

Health assessment comprises information related to the patient’s general health, such as (1) *Comorbidities*, which refer to co-occurring or additional disorders or diseases relative to a primary condition; (2) performance assessments, such as the *ECOG Performance Status* and *Karnofsky Performance Status*, used as health assessment tools to measure the functional statuses of patients; and (3) *History of Metastatic Cancer* (defined as a situation rather than a condition adhering to the mCODE specifications and Systematized Nomenclature of Medicine [SNOMED] classifications), which records the existence of a past episode of metastatic cancer.

Cancer treatment includes procedures (surgical and radiotherapy) and medication used to treat a patient with cancer with a specific treatment intent. They address a cancer condition or related conditions as the primary reason for treatment. While *a Surgical procedure* is a surgical action addressing a specific body site, *Radiotherapy* is a targeted treatment that also focuses on a particular area or volume in or on the body (*Body Site*) and is associated with a *Modality* (eg, electrons, photons, and high-dose rate) and *Technique* (eg, 3D, 2D, and intraoperative radiation therapy).

Outcomes involve tumor identification, tumor size, and disease status. *A tumor* is a *Body structure* used to identify tumors before removal from the body and specified by a location and laterality. References to the cancer condition and tumor morphology associated with this tumor are required. *Tumor Size* provides a mechanism for recording the dimensions of an identified tumor.

**Figure 4.**
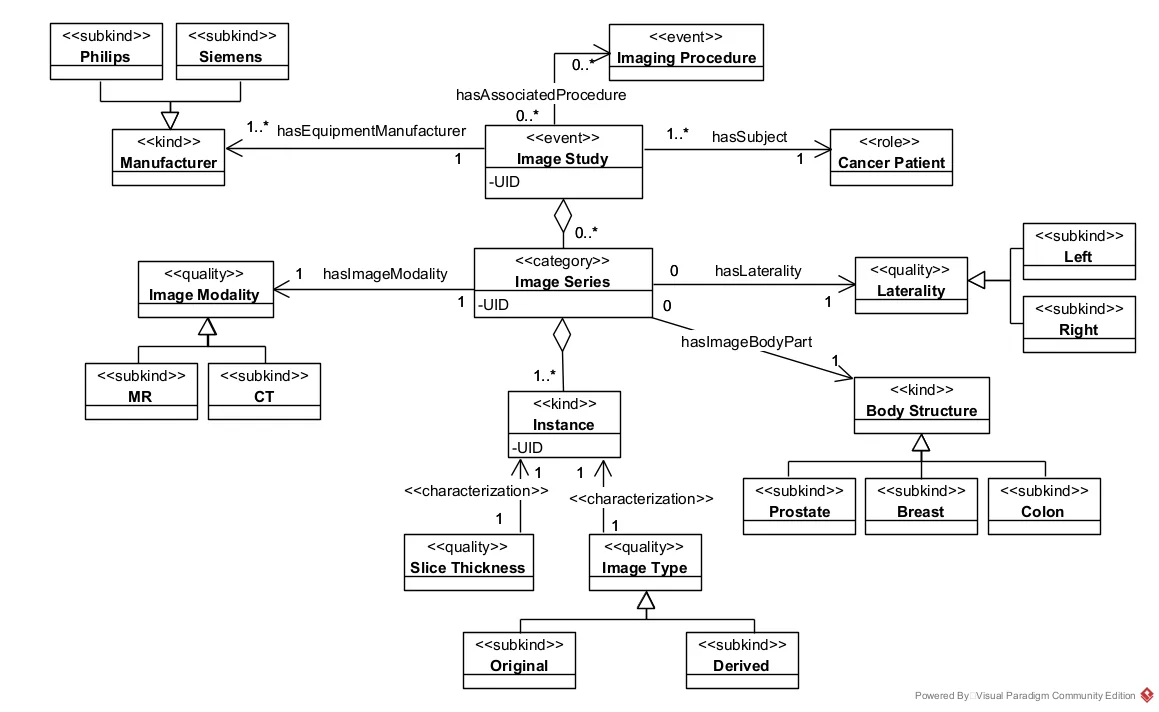
An illustration of part of the ontological conceptual model of the FHIR-based imaging aspects using OntoUML. CT: computed tomography; FHIR: Fast Healthcare Interoperability Resources; MR: magnetic resonance; UID: unique identifier. *Maximum number of occurrences, meaning many or unbounded.

For medical imaging, we ontologically analyzed the FHIR ImagingStudy [49] resource in the Diagnostic Medicine Module, which encapsulates the structure of a DICOM imaging study, including its associated series and imaging instances. In the DICOM standard, an image study represents a collection of medical images acquired for a specified clinical purpose or protocol (eg, a CT scan of the chest). Each image study comprises 1 or more image series of images acquired using the same imaging modality and targeting a specific anatomical region and laterality. We represented the imaging content with greater granularity using an ontological conceptual model with OntoUML stereotypes (Figure 4). In this model, *Image Study*, associated with an imaging procedure, is composed of 1 or more *Image Series,* performed on a *Cancer Patient,* and related to an equipment or device *Manufacturer* (eg, Siemens). Each *Image Series* is characterized by an *Imaging Modality* technique (eg, MR and CT), a *Body Structure* (eg, prostate, breast, and colon), and a specified *Laterality* (eg, left and right). Furthermore, each image instance in the series is characterized by *Slice Thickness* and *Image Type* (eg, original and derived).

Subsequently, we transformed the clinical and imaging conceptual models into machine-interpretable operational representations using the OWL 2 (Web Ontology Language 2) [50] formal language. This transformation is enabled by a terminology-binding phase that aligns core conceptual elements with standardized terminologies. The binding process, guided by mCODE and FHIR specifications, is performed manually under the expert’s supervision to ensure accurate and meaningful mapping.

Table 3 presents clinical-related concepts or elements and their corresponding mapped standard concepts (including direct and structural is-a mappings), with associated value sets defined by mCODE.

**Table 3.** Examples of mapping clinical concepts to standard terminologies.

Concept or element name	Mapping level	Standard source (vocabulary:code-label)
Primary Cancer Condition	is-a	SNOMED-CT:363346000^a^—Malignant neoplastic disease
Secondary Cancer Condition	is-a	SNOMED-CT:128462008—Metastatic malignant neoplasm
Body site	Direct	SNOMED-CT:123037004—Body structure and ICD-O-3^b^ codes
Tumor Marker Test	Direct	SNOMED-CT:250724005—Tumor marker measurement (mCODE^c^: “value set of LOINC^d^ codes and can be extended”)
Surgical Procedure	Direct	SNOMED-CT:387713003 (Surgical procedure (procedure))
Tumor	Direct	SNOMED-CT:52988006—Lesion

a SNOMED-CT: Systematized Nomenclature of Medicine Clinical Terms.

b ICD-O-3: International Classification of Diseases for Oncology, 3rd Edition.

c mCODE: Minimal Common Oncology Data Elements.

d LOINC: Logical Observation Identifiers Names and Codes.

Table 4 presents imaging-related concepts, their standard sources, and their mapping to DICOM. The concepts are standardized primarily using RadLex, SNOMED-CT, and Birnlex, and syntactically aligned with DICOM attributes via semantic annotations (*DICOM_Tag* and *DICOM*_*Name*).

**Table 4.** Examples of mapping imaging concepts to standard terminologies and DICOM^a^.

Concept name	Standard source (vocabulary:code)	DICOM attribute name (tag)
Image Modality	RadLex:RID10311^b^	Modality (0008,0060)
Laterality	RadLex:RID5821	Laterality (0020,0060)
Manufacturer	birnlex_12832	Manufacturer (0008,0070)
Body Structure	SNOMEDCT:52530000^c^	BodyPartExamined (0018,0015)
Slice Thickness	RadLex:RID28669	SliceThickness (0018,0050)

a DICOM: Digital Imaging and Communications in Medicine.

b RadLex: radiological lexicon.

c SNOMEDCT: Systematized Nomenclature of Medicine Clinical Terms.

### Formal Results

#### Overview

We present formal outcomes derived from the bottom-up strategy, integrated with core content, and managed using ontology management tools such as Protégé [51]. These outcomes provide an overview of key classes, object and data properties, and annotations. The hyperontology version 2.0 (December 2025) [40], represented using World Wide Web Consortium OWL 2 [50], defines more than 3900 classes, 9700 subclassOf relations, 130 equivalence definitions, 180 object properties, 60 data properties, 2200 exact match mappings to standard terminologies, and 730 query criteria.

#### Classes

The classes or concepts defined in the hyperontology are derived from AI4HI, standard concepts collected via semantic mappings to standards, core concepts from mCODE, and specific concepts required for semantic integration within EUCAIM. The hyperontology, as a controlled, structured vocabulary, standardizes these concepts by assigning each one a unique EUCAIM ID. These IDs serve as reference identifiers, enabling data interoperability among concepts represented with different uniform resource identifiers or codes across different terminologies. For instance, many identifiers are assigned for “Malignant neoplasm of breast,” including “254837009” in SNOMED and “C50” in ICD-10. Meanwhile, the hyperontology assigns a unique concept ID (CLIN1000060) to the clinical concept and establishes semantic mappings to different terminologies. This strategy helps unify heterogeneous data within EUCAIM, enabling consistent interpretation and exchange. Besides, EUCAIM IDs are meaningful identifiers that, using descriptive terms, reflect the semantic context of concepts across the different ontology modules. For instance, clinical and imaging concepts have EUCAIM IDs with “CLIN” and “IMG” prefixes, respectively. Although the concepts are separated into different modules, they are semantically interrelated through various relationships—for instance, MRI of breast (IMG1016224) is linked to Malignant neoplasm of breast (CLIN1000060) and Breast (CLIN1063727) via the “hasRelatedCondition” and “hasDirectProcedureSite” object properties, respectively. Additionally, the concepts are mapped using label-based exact-match similarity with various standard terminologies and vocabularies. For example, *Malignant neoplasm of breast* (CLIN1000060), defined from ICD-10 (C50), is mapped using *skos:exactMatch* to SNOMED-CT (254837009), ICD-O-3 (8000/3-C50.9), and NCIT (C4872). The matching process incorporates various synonyms and definitions from different sources. For instance, “Breast cancer” and “Malignant tumor of breast” are standard alternative labels of *Malignant neoplasm of breast* (Figure 6A and E).

In the context of EUCAIM, “standard sources” for various categories (eg, cancer types, body parts, histology or morphology, and laboratory tests) are specified by the experts. For instance, ICD-10 and ICD-O-3 are considered primary sources for cancer conditions (eg, *Malignant neoplasm of breast* and *Malignant neoplasm of ovary*) and body parts (eg, *breast* and *ovary*), respectively. However, these sources may lack explicit representation of certain essential or domain-specific concepts. For instance, cancer subtypes, such as “Malignant tumor of cecum,” “Carcinoma of breast,” “Primary malignant neoplasm of breast,” and “Primary malignant neoplasm of brain,” lack standard coding in ICD-10. This necessitates using alternative sources, such as SNOMED-CT, ICD-O-3, and NCIT, to fill the gap and ensure comprehensive representation across different levels of granularity. Similarly, multiple sources are defined for tumor marker tests, which are primarily determined from LOINC (following the mCODE specification) and expanded using OMOP Genomic.

To handle the heterogeneity of sources, accurate (taxonomic) semantic integration across different terminologies is required. On the syntactic level, domain-specific concepts are also mapped to OMOP and FHIR by establishing an alignment that covers the OMOP domains and IDs, as well as FHIR resources.

**Figure 5.**
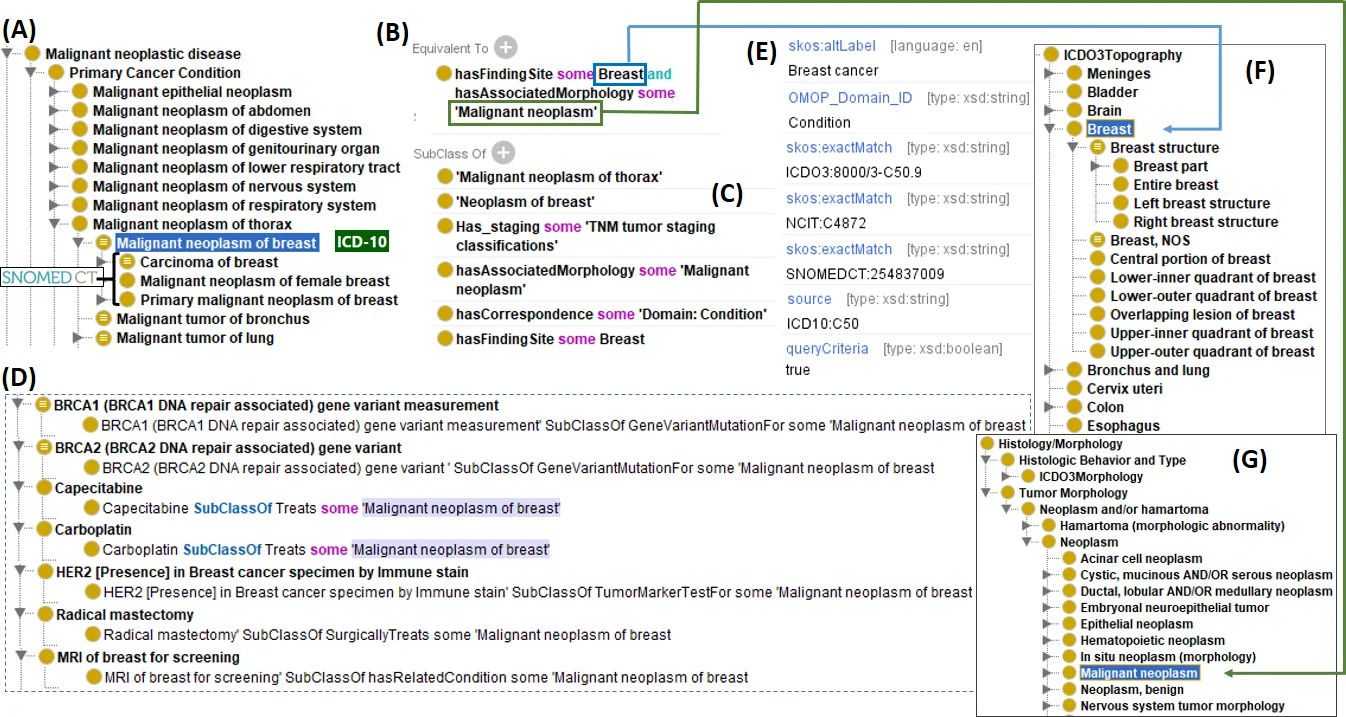
Part of the hyperontology around “Malignant neoplasm of breast,” represented using Protégé: (A) taxonomy of concepts, (B) semantic patterns, (C) restrictions, (D) concept usage, (E) semantic annotations, (F) taxonomy of Breast topography, and (G) taxonomy of morphology. BRCA1: breast cancer gene 1; BRCA2: breast cancer gene 2; HER2: human epidermal growth factor receptor 2; ICD-10: International Classification of Diseases, 10th Revision; ICDO3: International Classification of Diseases for Oncology, 3rd Edition; MRI: magnetic resonance imaging; NCIT: National Cancer Institute Thesaurus; NOS: not otherwise specified; OMOP: Observational Medical Outcomes Partnership; SNOMEDCT: Systematized Nomenclature of Medicine Clinical Terms.

Figure 5 illustrates a portion of the hyperontology surrounding the concept *Malignant neoplasm of breast*, focusing on (A) the structure of classes; (B) semantic patterns, such as equivalence, which define cancer conditions in terms of associated morphology and body site; (C) restrictions, such as associating the cancer type with cancer staging method, finding site, and histological types; (D) concept use that shows additional restrictions associating the cancer types with medication, surgical and imaging procedures, genetic mutations, and tumor marker tests; (E) annotations and mappings, such as alternative labels and exact match; (F) taxonomy of breast topography; and (G) taxonomy of tumor morphology.

**Figure 6.**
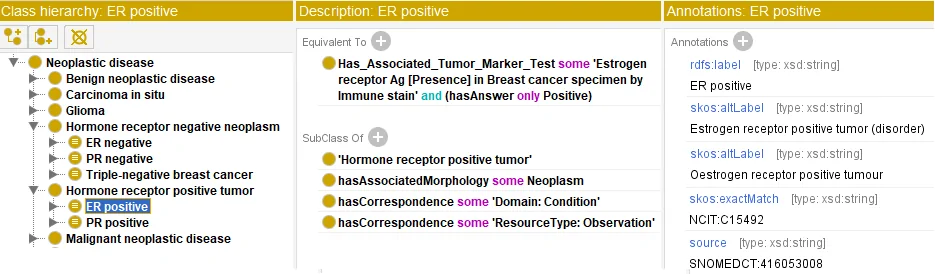
An illustration of the logical definition of ER positive, represented using Protégé. ER: estrogen receptor; PR: progesterone receptor; NCIT: National Cancer Institute Thesaurus; SNOMEDCT: Systematized Nomenclature of Medicine Clinical Terms.

Using Description Logic abstract syntax [52], we represent the equivalence semantic pattern applied to *Malignant neoplasm of breast* (Figure 6B), as follows:

- *Malignant neoplasm of breast* ≡ ∃hasFindingSite.Breast ⊓ ∃*hasAssociatedMorphology.”Malignant neoplasm”*

This pattern, adapted from SNOMED, formally provides the semantic meaning of breast cancer by explicitly associating the cancer type with the corresponding body part and morphology, using the object properties *hasFindingsite* and *hasAssociatedMorphology*, respectively.

#### Properties

##### Object Properties

The hyperontology specifies various object properties at different levels of granularity, considering the ORSD (specific detail), mCODE (generic level), and FHIR-based imaging (core level). Besides, relations have been reused from standard terminologies, such as SNOMED-CT (Table 5).

**Table 5.** Excerpt of object properties with their sources, definitions, as well as the domain and range.

Object property (source)	Definition	Domain	Range
*ComorbidityAssociatedWith* (ORSD^a^)	Associate various comorbid conditions (eg, *Abscess of liver*, *Cystadenoma of liver*, and *Intraabdominal bile collection*) with the related cancer types (eg, *Liver cancer*)	Disease	Malignant neoplastic disease
*GeneVariantMutationFor* (ORSD)	Link mutations or variants in genes (eg, *BRCA2^b^ [BRCA2 DNA repair associated] gene variant*) with the related cancer types (eg, *Breast cancer*)	Gene mutation	Malignant neoplastic disease
*Treats* (ORSD)	Specify a link between the drugs (eg, *floxuridine* and *leucovorin*) and the corresponding cancer types (eg, *Colon cancer*)	Drug or medicament	Malignant neoplastic disease
*SurgicallyTreats* (ORSD)	Establish a connection between the surgical procedures (eg, *Prostatectomy*) and the associated cancer types (eg, *Prostate cancer*)	Surgical procedure	Malignant neoplastic disease
*TumorMarkerTestFor* (ORSD)	Relate tumor marker tests (eg, *Cells.estrogen receptor/100 cells in Breast cancer specimen by Immune stain*) with the associated cancer types (eg, *Breast cancer*)	Pathology	Malignant neoplastic disease
*CancerStagingMethodFor* (ORSD)	Link the cancer staging classification methods (eg, *TNM*, *AJCC/UICC^c^*, and *FIGO^d^*) with the corresponding cancer types (eg, *Breast cancer* and *Ovarian cancer*)	Tumor staging	Malignant neoplastic disease
*CancerGradingMethodFor* (ORSD)	Associate grading systems (eg, *Gleason grading system for prostate cancer* and *Nottingham histologic grading system*) with the corresponding cancer types (eg, *Prostate cancer* and *Breast cancer*)	Histological grading systems	Malignant neoplastic disease
*AnswerOf* (ORSD)	Associate staging or grading values (eg, *Tumor regression Grade 1*, *cM1a*, and *GX*) with the corresponding systems or methods (eg, *Tumor regression grade*, *TNM Clin M*, and *Pathological grade*)	Qualifier	Staging and scales
*hasFindingSite* (SNOMED^e^)	Associate the cancer diagnosis (eg, *Malignant tumor of ascending colon*) with the finding location (eg, *Ascending colon*).	Malignant neoplastic disease	Body structure
*hasDirectProcedureSite* (SNOMED)	Link procedures (eg, “*MRI-US^f^ fusion guided prostate biopsy*” and *Prostatectomy*) with the corresponding body locations (eg, *Prostate*)	Procedure	Body structure
*hasAssociatedMorphology* (SNOMED) and *hasHistologyICDO* (SNOMED)	Establish a connection between the cancer types (eg, “*Malignant neoplasm of colon*” and “*Adenocarcinoma, NOS^g^, of cecum*”) and the associated histologic or morphologic types (eg, “*Malignant neoplasm*” and “*Adenocarcinoma, NOS*”)	Malignant neoplastic disease	Tumor morphology and histologic behavior and type
*hasBirthSex* (mCODE^h^)	Associate the patient with the corresponding birth sex (eg, *Female*, *Male*, and *Unspecified*)	Patient	Sex assigned at birth
*hasRelatedPrimaryCancerCondition* (mCODE)	Associate the secondary cancer condition with the primary cancer condition	Secondary Cancer Condition	Primary Cancer Condition
*hasFocus* (mCODE)	Link, at the generic level, the cancer stage and comorbidities with the associated primary cancer condition.	Tumor Staging Disease	Primary Cancer Condition
*hasTreatmentIntent* (mCODE)	Associate the treatment (eg, *Surgical procedure* and *Radiotherapy*) with the corresponding intent (eg, *Curative* and *Preventive*)	Treatment	Intents
*hasImageBodyPart* (FHIR^i^)	Link the image series with the corresponding body part (eg, *Lung*, *Breast*, and *Brain*)	Image series	Body structure
*hasImageModality* (FHIR)	Link the image series with the corresponding image modality (eg, *MR^j^* and *CT^k^*)	Image series	Imaging modality

a ORSD: Ontology Requirements Specification Document.

b BRCA2: breast cancer gene 2.

c AJCC/UICC: American Joint Committee on Cancer/Union for International Cancer Control.

d FIGO: International Federation of Gynecology and Obstetrics.

e SNOMED: Systematized Nomenclature of Medicine.

f MRI-US: magnetic resonance imaging–ultrasound.

g NOS: not otherwise specified.

h mCODE: Minimal Common Oncology Data Elements.

i FHIR: Fast Healthcare Interoperability Resources.

j MR: magnetic resonance.

k CT: computed tomography.

##### Data Properties

Various data properties have been specified in the hyperontology, primarily derived from mCODE and FHIR-based imaging (Table 6).

##### Annotation Properties

The hyperontology specifies and reuses various annotations to fulfill mapping requirements and facilitate the ontology’s usability across different applications, such as federated queries and ETL processes (Table 7).

**Table 6.** Excerpt of data properties with their sources, definitions, as well as the domain and range.

Data property (source)	Definition	Domain	Range
*PatientIdentifier* (mCODE^a^)	An identifier of the patient	Patient	xsd:string
*AssertedDate* (mCODE)	The date the cancer condition (primary or secondary) was first asserted	Cancer Condition	xsd:dateTime
*Deceased* (mCODE)	The vital status of the patient	Patient	xsd:boolean
*ImageStudyUID* (FHIR^b^)	A unique identifier for the image study	Image Study	xsd:string

a mCODE: Minimal Common Oncology Data Elements.

b FHIR: Fast Healthcare Interoperability Resources.

**Table 7.** Excerpt of semantic annotations with definitions and some examples.

Annotation	Definition	Example
*skos:prefLabel*	A lexical label that represents the preferred label associated with a concept. Preferred labels are derived from the standard sources.	The preferred labels for the primary cancer types and body sites are those defined in ICD-10^a^ (eg, *Malignant neoplasm of breast* and *Malignant neoplasm of colon*) and ICD-O-3^b^ (eg, *Breast* and *Colon*).
*skos:altLabel*	A lexical label that represents the alternative labels, or synonyms, associated with a concept. They are collected through mappings with standard terminologies or vocabularies.	*Breast cancer* and *Malignant tumor of breast* are alternative labels of *Malignant neoplasm of breast.*
*skos:exactmatch*	A mapping property used to link 2 concepts, indicating a high degree of confidence that the concepts can be used interchangeably across information retrieval applications.	The *exact match* of ICD10:C18 (malignant neoplasm of colon) is SNOMEDCT:363406005^c^ (malignant tumor of colon).
*source*	An EUCAIM^d^-specific property to specify the source vocabulary and code of concepts. Domain experts determine vocabulary selection. For instance, ICD-10, ICD-O-3, and RadLex^e^ are defined as standard sources for main cancer types, body parts, and medical imaging, respectively.	ICD10:C50 and ICDO3:C50 are the standard source codes for *Malignant neoplasm of breast* and *Breast*, respectively. The standard source codes for imaging modalities, such as MR^f^ and CT^g^, are RID10312^h^ and RID10321, respectively.
*queryCriteria*	An EUCAIM-specific property to specify the query criteria for the federated querying.	Key categories, such as cancer types (eg, *Malignant neoplasm of breast* and *Malignant neoplasm of colon*), body sites (eg, *Breast* and *Colon*), and image modalities (eg, *MR* and *CT*), are specified as query criteria.
*OMOP_Concept_Name*	Syntactic mapping with OMOP^i^ to specify the concept name.	*Prostate specific antigen measurement.*
*OMOP_Concept_code*	Syntactic mapping with OMOP to specify the concept code.	*63476009.*
*OMOP_Vocabulary_ID*	Syntactic mapping with OMOP to specify the main source vocabulary.	*SNOMED^j^.*
*OMOP_Domain_ID*	Syntactic mapping with OMOP to specify the domain ID.	*Measurement.*
*FHIR_ResourceType*	Syntactic mapping with FHIR^k^ to specify the resource type.	*Observation.*
*DICOM_Name*	Syntactic mapping with DICOM^l^ to specify the DICOM name.	*Imaging modality* is aligned with the “Modality” DICOM name.
*DICOM_Tag*	Syntactic mapping with DICOM to specify the DICOM tag.	*Imaging modality* is aligned with (0008,0060) DICOM tag.

a ICD-10: International Classification of Diseases, 10th Revision.

b ICD-O-3: International Classification of Diseases for Oncology, 3rd Edition.

c SNOMEDCT: Systematized Nomenclature of Medicine Clinical Terms.

d EUCAIM: Cancer Image Europe.

e RadLex: radiological lexicon.

f MR: magnetic resonance.

g CT: computed tomography.

h RID: RadLex identifier.

i OMOP: Observational Medical Outcomes Partnership.

j SNOMED: Systematized Nomenclature of Medicine.

k FHIR: Fast Healthcare Interoperability Resources.

l DICOM: Digital Imaging and Communications in Medicine.

### Logical Definitions for Data Harmonization

#### Overview

Incorporating logical definitions is increasingly being adopted in ontology development to support semantic interoperability and data integration [53]. With the support of clinical experts, various logical definitions have been created to clarify the semantic meaning of specific classes. These definitions, which act as simple design patterns, are used to explicitly and precisely define complex, ambiguous, or nonstandardized concepts in terms of other standard atomic concepts. By applying these definitions, logical consequences can be generated using a logic reasoner (eg, HermiT [54]), thereby inferring the position and classification of concepts within the ontology’s hierarchy [53].

#### Tumor Marker Test Results

For breast cancers sensitive to estrogen (ER) and progesterone (PR) receptors or biomarkers, tumor marker test results are often reported as hormone receptor status, classified as ER-positive (ER+), ER-negative (ER−), PR-positive (PR+), and PR-negative (PR−). These statuses have been interpreted and standardized in 2 ways (Table 1): (1) as conditions (disorders; eg, *ER positive* (SNOMED-CT, 416053008) and *PR negative* (SNOMED-CT, 441118006) tumor) or (2) tumor marker tests (eg, *ER* (LOINC, 85337‐4) and *PR* (LOINC, 85339‐0)) associated with their results (eg, *Positive* (SNOMED-CT, 10828004) or *Negative* (SNOMED-CT, 260385009)). To ensure data harmonization, a logical definition is created, informed by clinical expertise, to precisely define hormone receptor statuses as specific attributes related to breast cancer types and clarify their association with corresponding tumor marker tests and results. In Figure 6, the term *“ER-positive”* is defined logically. A formal description of this definition is given as follows:

- *ER positive* ≡ ∃*Has_Associated_Tumor_Marker_Test*.”*Estrogen receptor Ag [Presence] in Breast cancer specimen by Immune stain”* ⊓ ∀*hasAnswer.Positive*.

While the object property *Has_Associated_Tumor_Marker_Test* connects cancer types with their associated tumor marker tests, *hasAnswer* links these tests with their corresponding qualitative results (eg, positive and negative). By creating this definition, we resolve data heterogeneity across data holders by harmonizing the interpretations of tumor marker test results.

#### Molecular Breast Cancer Subtypes

**Figure 7.**
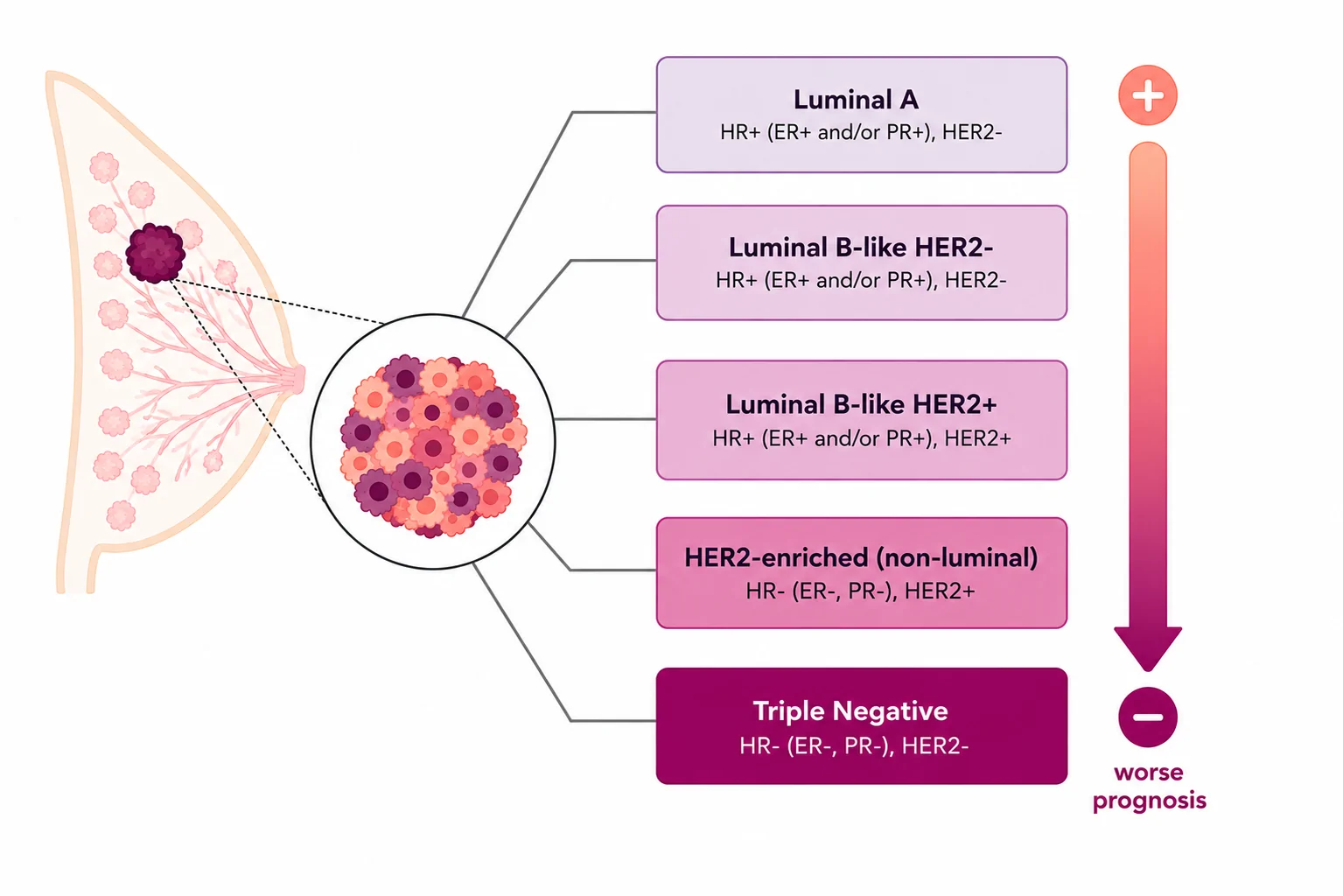
Characteristics of the molecular subtypes of breast cancer (adapted from Attalla et al [57], which is published under the Creative Commons Attribution 4.0 International License [58]). ER: estrogen receptor; HER2: human epidermal growth factor 2; HR: hormone receptor; PR: progesterone receptor.

Various subtypes of breast cancer have been defined by AI4HI (Table 2), including molecular subtypes (eg, Luminal A, Luminal B (HER2-negative or HER2-positive), HER2-enriched/HR-negative (non-luminal), and Triple Negative or Basal-like (all HR and HER2-negative)). These subtypes lack a clear semantic representation in standard terminologies. While Luminal A and Luminal B are defined in NCIT (under Breast *Carcinoma by Gene Expression Profile* (C53553)), specific types of Luminal B (HER2 negative or HER2 positive) and HER2-enriched do not have formal definitions in coding systems. Nevertheless, molecular subtypes can be interpreted and classified based on the presence or absence of specific hormone receptors (HRs) and proteins [55-57]: ER (Estrogen Receptor), PR (Progesterone receptor), HER2 (Human Epidermal growth factor Receptor 2), and Ki67 (Cell proliferation marker). Figure 7 illustrates the classification criteria for these subtypes, which have been validated by oncology and pathology experts.

For example, *Luminal B-HER2 negative* and *HER2 positive (enriched)/HR negative (non-luminal*) exhibit the following distinguishing features:

- “Luminal B-HER2 negative” is interpreted as a combination of HER2 negative, ER positive, and/or PR positive, and Ki67 positive.
- “HER2 positive (enriched)/HR negative (non-luminal)” is interpreted as a combination of HER2 positive, ER negative/absent, and PR negative/absent.

In the hyperontology, we explicitly defined these molecular subtypes by integrating diverse features. The logical definitions of *Luminal B-HER2 negative and HER2 positive (enriched)/HR negative (non-luminal*) are given as follows:

- *Luminal B-HER2 negative* ≡ *HER2 negative* ⊓ (*ER positive* ⊔ *PR positive*) ⊓ *MKI67 positive*
- *HER2 positive (enriched)/HR negative (non-luminal*) ≡ *HER2 positiv*e ⊓ *ER negative* ⊓ *PR negative*

Consequently, breast cancer molecular subtypes are standardized, classified, and incorporated into the hierarchy (by inference) at precise levels of granularity.

**Figure 8.**
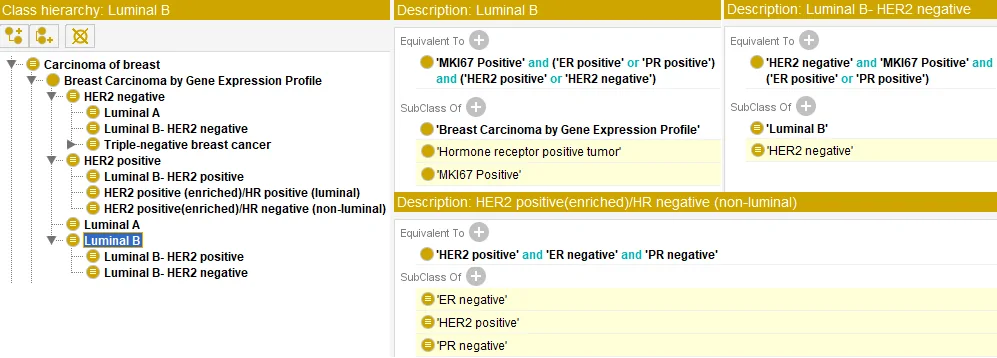
An illustration in Protégé of the semantic pattern defining “Luminal B-HER2 negative” and “HER2 positive (enriched).” ER: estrogen receptor; HER2: human epidermal growth factor 2; HR: hormone receptor; PR: progesterone receptor.

Figure 8 illustrates 3 different logical definitions (Luminal B, Luminal B-HER2 negative, and HER2 positive (enriched)/HR negative (non-luminal)) and the inferred classifications of concepts (yellow background) using HermiT [54].

### Ontology Evaluation and Validation

#### Overview

This section discusses the results achieved by applying the multidimensional evaluation and validation strategy. For consistency, we used the logic reasoner HermiT [54] (version 1.4.3), an OWL 2 inference engine, to check for logical errors and inconsistencies and verify the correct inference of implicit knowledge. For the requirement-driven evaluation, methods such as ORSD verification, ontology revision with expert help, and ontology testing by formalizing generic CQs using SPARQL were used. Finally, to validate the hyperontology’s applicability, reusability, and extensibility, ontology population with real-world scenarios from AI4HI and ontology extension with new datasets have been performed.

#### ORSD Verification

ORSD verification involves experts who did not participate in the ontology development process to ensure that the ontology meets the content or quality requirements documented in the ORSD. Specifically, the evaluation process focuses on the following steps:

- CQ validation: This step includes (1) confirming that the CQs defined in the ORSD fully capture the clinical and imaging knowledge provided by AI4HI and (2) checking whether the ontology faithfully represents and describes the clinical and imaging concepts listed in the ORSD as answers to the CQs. For example, the body parts corresponding to the general CQ5, “What are the main body sites affected by cancer?” as shown in Textbox 1, are reviewed for accuracy.
- Terminological accuracy review: Assessing the ontology with particular attention to standardized labels specified by the use cases to ensure terminological consistency and correctness.
- Standards and terminologies integration: Verifying the correct incorporation of standards and terminologies, such as LOINC and SNOMED-CT for patient sex, by evaluating semantic annotations, including labels, sources, syntactic mappings, and semantic properties such as equivalence, thereby ensuring both semantic and syntactic interoperability.
- Documentation of observations: Comprehensive reports documenting findings were produced. Such reports were compiled to summarize coverage status, reference specific ontology elements, and detail any gaps or issues that arose. Recommendations from this review informed refinement cycles of the ontology.

This multistep, expert-driven approach ensures transparency, reproducibility, and a comprehensive assessment of how effectively the EUCAIM hyperontology meets its stated requirements.

#### Ontology Revision With Experts

Ontology revision is a collaborative process involving domain experts, such as oncologists, radiologists, and pathologists, who can help ensure that the hyperontology accurately reflects the domains of oncology and medical imaging. Throughout multiple meetings with the experts, we systematically revised and evaluated the ontology content, considering concept classification, semantic interactions, and consistency of standard terms. We asked for feedback on different questions, such as: (1) Are the generic categories semantically organized? (2) Are there cancer subtypes that should be included in the hierarchy? (3) Are the terms used in the ontology consistent from a clinical perspective? (4) Should any terms be redefined or harmonized with widely accepted standards? (5) Are the semantic relationships accurately represented? (6) Are any other relationships required?

Based on the experts’ feedback, we adjusted the classification system to include new classes or refine the hierarchy. For instance, cancer subtypes, such as cancer of the breast parts (eg, nipple and areola), have been included in the taxonomy under *Malignant neoplasm of breast* (ICD-10, C50). Additionally, classifying tumor marker tests has required expert intervention, especially for tests not included in the mCODE terminology binding [29], which covers tests defined in LOINC (eg, *Estrogen receptor Ag [Presence] in Breast cancer specimen by Immune stain* (85337‐4)). For instance, a specific verification was required for tumor marker tests for brain cancer (eg, *IDH1*, *P53*, *ATRX*, and *MGMT*), which are represented as genetic tests in OMOP Genomic. In contrast, mCODE distinguishes between tumor marker tests and genetic tests.

The experts have also helped expose erroneous assignments of concepts that were incorrectly mapped or standardized throughout the ETL process and provided as input for the ORSD. For instance, the term “breastfeeding,” which is required for assessing breast cancer risk factors, is mistakenly assigned to *Breastfed* (SNOMED-CT, 169741004), an infant feeding method, rather than to *Breastfeeding* (SNOMED-CT, 413712001), a breast function related to the mother. Another example is related to the degrees of relatives with cancer (*First degree*/*Second degree*), which are relevant for assessing genetic or familial cancer risk. These values or concepts are incorrectly mapped to severity degrees in SNOMED-CT (264500008/263868004) instead of blood relative degrees: *First degree blood relative* (SNOMED-CT, 125678001) and *Second degree blood relative* (SNOMED-CT, 699110007). These values are required to semantically define the family history findings (eg, *Family history of malignant neoplasm of breast in first degree relative*). Accordingly, the relevant concepts have been included in the ontology model.

#### Formalization of CQs Using SPARQL

To assess whether the hyperontology effectively covers the application domain and meets content or quality requirements, CQs have been formalized as SPARQL [48] queries that the ontology should answer. These queries help validate the hyperontology at the structure or schema (terminological box) level, focusing on ontology classes, relationships, hierarchies, restrictions, and axioms. Table 8 presents examples of SPARQL queries that translate some of the generic CQs previously described in this study. In addition to the prefixes used for SPARQL validation at the data level, *owl* [59] is required for schema-level validation.

**Table 8.** Examples of SPARQL^a^ queries translating generic competency questions (CQs) using restrictions.

CQ	SPARQL query	Result, n	Example of answers (EUCAIM^b^ ID)
CQ2. What are the main types and subtypes of cancer conditions identified during the cancer diagnosis?	SELECT DISTINCT ?cancer_condition WHERE { ?cancer_patient rdfs:subClassOf [ a owl:Restriction ; owl:onProperty eucaim:diagnosedWith ; owl:someValuesFrom ?generic_cancer_condition ]. OPTIONAL { ?cancer_condition rdfs:subClassOf* ?generic_cancer_condition . }}	268	Malignant neoplasm of prostate (CLIN1000075) Malignant tumor of meninges (CLIN1054131) Non-Hodgkin’s lymphoma (clinical) (CLIN1046323)
CQ5. What are the main body sites affected by cancer?	SELECT DISTINCT ?cancer_condition ?body_site WHERE { ?cancer_condition rdfs:subClassOf [ a owl:Restriction ; owl:onProperty eucaim:hasFindingSite ; owl:someValuesFrom ?body_site ]. }	213	Prostate (BP1000021) Esophagus (BP1000303) Rectum (CLIN1063724)
CQ6. Have any comorbidities been identified during the diagnostic process?	SELECT DISTINCT ?cancer_condition ?comorbidity WHERE { ?comorbidity rdfs:subClassOf [ a owl:Restriction ; owl:onProperty eucaim:ComorbidityAssociatedWith ; owl:someValuesFrom ?cancer_condition ]. }	42	Echinococcosis of liver (CLIN1045766) Hemangioma (CLIN1046540)
CQ17. Which imaging assessment methods are used in cancer diagnosis?	SELECT DISTINCT ?imaging_assessment_method WHERE { ?imaging_assessment_method rdfs:subClassOf [ a owl:Restriction ; owl:onProperty eucaim:ImagingAssessmentMethodFor ; owl:someValuesFrom ?cancer_condition ]. }	3	PI-RADS^c^ assessment (IMG1005457) BI-RADS^d^ assessment (IMG1005459)
CQ23. Which medication or drug therapy is used for cancer treatment?	SELECT DISTINCT ?cancer_condition ?medication WHERE { ?medication rdfs:subClassOf [ a owl:Restriction ; owl:onProperty eucaim:Treats ; owl:someValuesFrom ?cancer_condition ]. }	66	Capecitabine (CLIN1035836) Chemotherapy cycle (CLIN1035191)

a SPARQL: SPARQL Protocol and Resource Description Framework Query Language.

b EUCAIM: Cancer Image Europe.

c PI-RADS: Prostate Imaging Reporting and Data System.

d BI-RADS: Breast Imaging Reporting and Data System.

#### Ontology Population Using Real-World Scenarios

To validate the applicability of the hyperontology, real-world breast and prostate cancer scenarios (in unstructured text) were collected from AI4HI to enrich and populate the hyperontology. We manually identified and extracted instances (individuals) and assigned them to the corresponding predefined classes or concepts. Semantic relationships are also maintained among the individuals, considering the object properties defined in the ontology model. The hyperontology is also used as a knowledge base, with its semantic content accessed via SPARQL queries. In what follows, we present 2 scenarios provided by ChAImeleon (breast cancer) and ProCAncer-I (prostate cancer).

In the breast cancer scenario, a female patient underwent various procedures, including *mammography, ultrasound*, and *core needle biopsy of breast*. These procedures informed multiple diagnostic outcomes, including tumor diagnosis of *Ductal Carcinoma grade II*, imaging assessments (eg, *5*), clinical staging (eg, *cT2* and *cN0*), and tumor marker test results (eg, *ER-positive* and *PR-positive*). Additionally, a *hypofractionated stereotactic radiotherapy* was administered, resulting in a *complete response*. In the prostate cancer scenario, the diagnosis of a male patient begins with prostate-specific antigen (PSA) laboratory tests and a *digital rectal examination* procedure. It progresses through imaging techniques, such as *multiparametric MRI* and *fusion biopsy*, followed by a *prostatectomy* surgical procedure. Diagnostic interpretations included imaging observations (eg, *PI-RADS5*), histological grading (eg, *Gleason score 4+3*), clinical staging (eg, *cT2b* and *cN0*), and pathological staging (eg, *pT2* and *pN0*). Tumor maximum dimensions and volume were also considered in the diagnostic process (Textbox 2).

Textbox 2. **Real-world breast and prostate cancer scenarios.**

**Breast cancer scenario**

A mammography and ultrasound were performed on a 59-year-old female patient born in February 1957, which detected a lump in her breast. The ultrasound indicated a suspicious lesion, most probably cancer (BI-RADS 5). For that reason, a core needle biopsy of breast was performed 6 weeks later, confirming the suspicion and diagnosing her with Ductal Carcinoma grade II, cT2N0, ER-positive, PR-positive, HER2-negative, and Ki67 at 12%. A thorax, abdomen, and pelvis CT scan 2 weeks after the biopsy showed no evidence of metastatic disease, confirming the clinical stage IIA (cT2N0M0). The patient received neoadjuvant chemotherapy starting 1 month after the CT scan. A radical mastectomy was performed 6 months after the chemotherapy, and no residual tumor was found (pT0N0). Another thorax, abdomen, and pelvis CT scan was performed 3 weeks after surgery, showing no evidence of metastatic disease (M0). Six weeks after surgery, the patient began hypofractionated stereotactic radiotherapy and achieved a complete response.

**Prostate cancer scenario**

The patient is a 59-year-old male with a PSA value of 7.16 (ng/mL) and a free PSA of 5.04 (ng/mL). The patient had a positive digital rectal examination, and the urologist sent him to perform a multiparametric MRI. The MRI performed 22 days after the PSA laboratory test was deemed positive and revealed a PI-RADS 5 lesion, with a maximum diameter of 10 mm, located in the right peripheral zone basal posterolateral region, with a clinical stage of cT2b and cN0. The patient underwent a fusion biopsy, which revealed a cT2 cancer stage. Because of the positive findings, the patient was referred to undergo a prostatectomy. The prostatectomy results also confirmed the positive findings, revealing a 4+3 Gleason score lesion with an overall volume of 0.7 cc, a maximum diameter of 17 mm, and a stage of pT3b, pN0, and intraductal carcinoma. After 6 months, MRI and PET examinations were performed, where liver metastasis was identified with reported stage cNX, cM1c.

**Figure 9.**
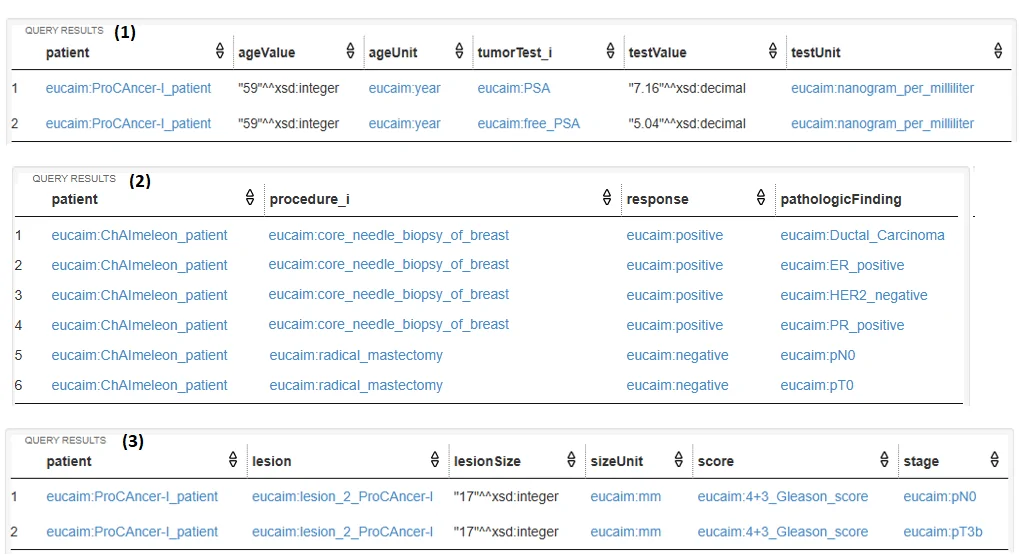
An illustration of the query results using the Apache Jena Fuseki server. ER: estrogen receptor; HER2: human epidermal growth factor 2; PR: progesterone receptor; PSA: prostate-specific antigen.

We manually mapped the various information to their corresponding semantic references (classes and properties), thereby populating the hyperontology with real-world data. This enabled the logic reasoner (HermiT) to infer the complete diagnosis, including the imaging findings and clinical or pathological interpretation results. Furthermore, we considered these results to query the populated ontology for cancer diagnosis information, thereby demonstrating its applicability and effectiveness as a knowledge base for extracting semantic content. Table 9 presents 3 queries to validate the hyperontology at the individual- or data-level (assertional box). We defined them considering *query criteria* specified in EUCAIM (eg, sex, age, cancer type, body part, staging/grading, tumor marker test or result, and clinical or imaging procedure). The queries are implemented using SPARQL [48], Owlready2 (a Python library for managing Ontology Web Language ontologies) [60], and Apache Jena Fuseki (a SPARQL server) [61]. The following prefixes are required for the query execution: *rdf* [62], *rdfs* [63], and *eucaim* [64]. Figure 9 illustrates the query results obtained by running a local Fuseki server.

**Table 9.** Queries syntax and their SPARQL^a^ translation.

Query syntax	SPARQL translation
Query (1): Cancer patients (COM1001051) older than 55 years (COM1000151) and have completed a *PSA^b^* (CLIN1000227) tests with a level greater than 5 ng/mL (COM1000156)	*SELECT ?patient ?ageValue ?ageUnit ?tumorTest_i ?testValue ?testUnit WHERE {* *?patient rdf:type eucaim:COM1001051* . *?patient eucaim:hasAgeAtDiagnosis ?age . ?age eucaim:Value ?ageValue* . *?age eucaim:hasUnitofTime ?ageUnit* . ?tumorTest rdfs:subClassOf* eucaim:CLIN1000227 . ?tumorTest_i rdf:type ?tumorTest . *?patient eucaim:Is_Subject_For ?tumorTest_i . ?tumorTest_i eucaim:Value ?testValue* . *?tumorTest_i eucaim:hasUnitofMeasure ?testUnit . ?testUnit rdf:type eucaim:COM1000156* . *} HAVING (?ageValue > 55 && ?ageUnit = eucaim:year && ?testValue > 5 && ?testUnit = eucaim:nanogram_per_milliliter*)
Query (2): Cancer patients (COM1001051) who are *female* (COM1001370) and have undergone a *removal procedure* (CLIN1058186) with the associated response and pathologic findings	*SELECT ?patient ?procedure_i ?response ?pathologicFinding WHERE {* *?patient rdf:type eucaim:COM1001051* . *?patient eucaim:hasBirthSex ?sex . ?sex rdf:type eucaim:COM1001370* . ?procedure rdfs:subClassOf* eucaim:CLIN1058186 . ?procedure_i rdf:type ?procedure . *?patient eucaim:hasUndergone ?procedure_i . ?procedure_i eucaim:hasResult ?response* . *?procedure_i eucaim:Has_pathologic_interpretation_result ?pathologicFinding . }*
Query (3): Cancer patients (COM1001051) found to have a lesion (IMG1016402) with a maximum dimension greater than 10 mm, and return the related score and stage	*SELECT ?patient ?lesion ?lesionSize ?sizeUnit ?score ?stage WHERE {* *?patient rdf:type eucaim:COM1001051 . ?lesion rdf:type eucaim:IMG1016402* . *?lesion eucaim:hasAssociatedPatient ?patient* . *?lesion eucaim:MaximumDimensionValue ?lesionSize . ?lesion eucaim:hasUnitofDiameter ?sizeUnit* . *?lesion eucaim:hasScore ?score . ?lesion eucaim:hasStage ?stage* . *} HAVING (?lesionSize >10 && ?sizeUnit = eucaim:mm*)

a SPARQL: SPARQL Protocol and Resource Description Framework Query Language.

b PSA: prostate-specific antigen.

#### Ontology Enrichment

The enrichment process aims to validate the extendibility of the hyperontology. Additional cancer types (n=17) have been incorporated by leveraging the contributions of new data providers (n=12) who have joined the EUCAIM platform. Examples of newly provided cancer types are thyroid, brain, ovarian, bladder, gallbladder, stomach, bone, and pancreatic cancers. The contributions integrate various types of clinical and imaging information, including cancer types, histological subtypes, laboratory or tumor marker tests and results, cancer staging or scoring, surgical or imaging procedures, and imaging modalities. Moreover, additional information is incorporated for cancer types already included in the hyperontology. For example, in the case of prostate cancer, *PI-RADS* is specified in the hyperontology as the standard method for interpreting prostate magnetic resonance imaging (MRI). However, data holders have also provided alternative assessment methods, such as the *Likert scoring system* [65], which reflect the radiologist’s expert interpretation. In addition, for prostate cancer, additional clinical procedures have been considered, such as *Systematic Prostate Biopsy* and *Targeted Prostate Biopsy*, defined in NCIT, enhancing the scope of removal procedures. For imaging, new modalities and techniques, such as *Whole Slide Imaging (WSI)* and *Digitized hematoxylin-eosin (HE) slides*, have been introduced, expanding the scope to encompass digital pathology imaging in conjunction with radiology techniques (eg, *MRI and CT*).

Regarding data formatting, the contributions are presented as sets of labels or values associated with variables defined within each data provider’s dataset. These labels, which are not standardized, exhibit some ambiguity or incompleteness, making data analysis and, thereby, the enrichment process challenging. In this regard, a structured process is required to ensure the efficient semantic integration and harmonization of information. Three main phases have been conducted throughout the enrichment process: (1) data analysis and extraction, (2) semantic mapping, and (3) ontology integration.

**Figure 10.**
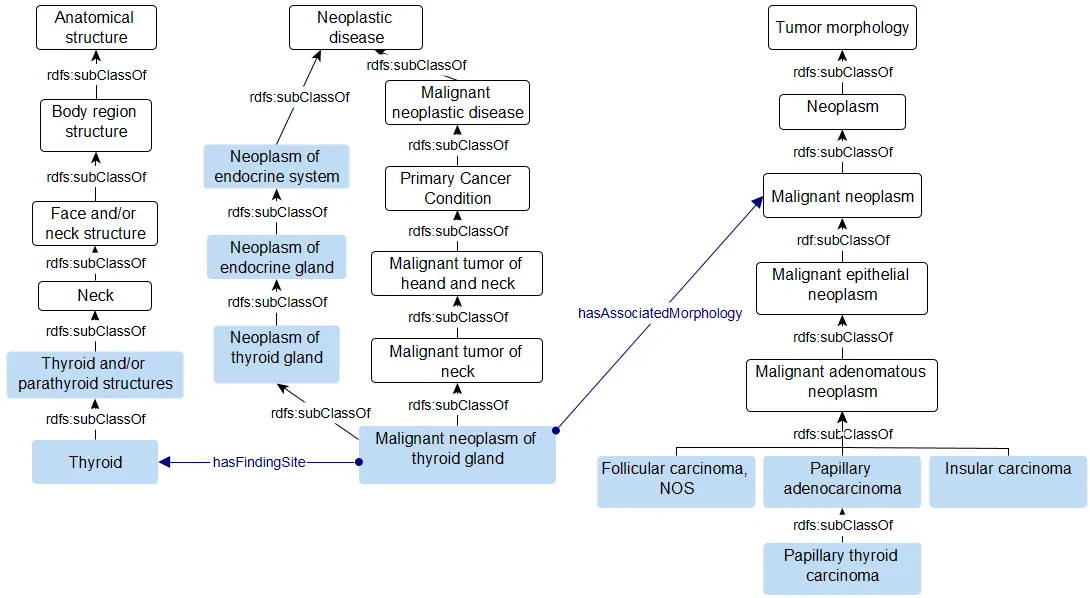
An illustration of ontology enrichment—integration of new concepts (blue background)—around thyroid cancer. NOS: not otherwise specified.

Table 9 presents an example of a new cancer type, thyroid cancer, with some clinical or biological and imaging information. Except for the cancer staging values (eg, N0 and N1a) and imaging modality (eg, scintigraphy), which are defined in previous versions of the hyperontology, the provided clinical and biological data are considered for enrichment and integration. Due to incomplete information, mapping to standards and terminologies is challenging and requires manual review. For instance, histological subtypes, such as “papillary” and “follicular,” can be mapped to various concepts from the OMOP *Observation* domain. The former is mappable to “Papillary carcinoma, NOS” (ICDO3, 8050/3), “Papillary adenocarcinoma, NOS” (ICDO3, 8260/3), and “Papillary thyroid carcinoma” (SNOMED-CT, 1336196002). Meanwhile, the latter maps to “Follicular carcinoma, NOS” (ICDO3, 8330/3) and “Follicular adenocarcinoma” (SNOMED-CT, 5257006). In this regard, the involvement of experts is required to clarify the terms, preventing any loss of granularity or precision during the mapping process. Figure 10 illustrates part of the ontology integration phase for thyroid cancer concepts (blue background), including cancer and histological subtypes, as well as body parts.

New standard concepts, such as *Malignant neoplasm of thyroid gland*, *Thyroid*, and *Insular carcinoma* (mapped to *Poorly differentiated thyroid carcinoma*; Table 10), have been integrated into the taxonomy and interconnected using semantic relations already defined in the ontology model (eg, *hasFindingsite* and *hasAssociatedMorphology*). Meanwhile, some additional taxonomic mappings have been conducted and incorporated into the hierarchy to enhance the semantic classification of terms. For instance, *Malignant neoplasm of thyroid gland*, classified as *Malignant neoplasm of neck*, is also a specification of *Neoplasm of thyroid gland* in SNOMED, introducing a new branch of concepts into the hyperontology’s taxonomy.

**Table 10.** Examples of clinical or biological and imaging data provided by new data holders (thyroid cancer).

Category and label or term as provided by data holder	Standard concept (semantic mapping-direct)	Standard source (semantic mapping-direct)
Cancer type		
Thyroid cancer	*Malignant neoplasm of thyroid gland*	ICD10:C73^a^
Histological subtype		
P (papillary)	*Papillary adenocarcinoma, NOS^b^*	ICDO3:8260/3^c^
FVP (follicular variant of papillary thyroid carcinoma)	*Papillary carcinoma, follicular variant*	ICDO3:8340/3
F (follicular)	*Follicular carcinoma, NOS*	ICDO3:8330/3
HC (Hurthle cell)	*Oxyphilic adenocarcinoma*	ICDO3:8290/3
IC (insular carcinoma)	*Poorly differentiated thyroid carcinoma*	ICDO3:8337/3
TVP (trabecular variant papillary)	*Trabecular adenocarcinoma*	ICDO3:8190/3
Laboratory test		
Thyroglobulin test	*Thyroglobulin [Mass/volume] in Serum or Plasma*	LOINC:3013‐0^d^
Thyroglobulin antibody test	*Thyroglobulin Ab [Units/volume] in Serum or Plasma*	LOINC:8098-6
Thyrotropin test	Thyrotropin [Units/volume] in Blood	LOINC:3015-5
Stimulated thyroglobulin test	N/A^e^	N/A
Stimulated thyroglobulin antibody test	N/A	N/A
Cancer staging method		
8th edition of the American Joint Commission on Cancer (AJCC)/TNM staging system for thyroid cancer	*AJCC/UICC^f^ 8th edition*	CancerModifier:8th_AJCC/UICC
Cancer staging category or value		
pN category	pN	SNOMEDCT:371494008^g^
N0	pN0	NAACCR:890@p0^h^
N1a	pN1a	NAACCR:890@p1A
Imaging modality		
Scintigraphy	Scintigraphy	RadLex:RID34428^i^

a ICD10: International Classification of Diseases, 10th Revision.

b NOS: not otherwise specified.

c ICDO3: International Classification of Diseases for Oncology, 3rd Edition.

d LOINC: Logical Observation Identifiers Names and Codes.

e N/A: not applicable.

f UICC: Union for International Cancer Control.

g SNOMEDCT: Systematized Nomenclature of Medicine Clinical Terms.

h NAACCR: North American Association of Central Cancer Registries.

i RadLex: radiological lexicon.

Table 11 presents some metrics for new concepts and relationships compared to those for reused ones. Extending and enriching the hyperontology, primarily at the domain-specific level, has broadened its scope, making it more applicable and valuable for querying, annotation, and segmentation applications and supporting the ETL process. Besides, the extension helped assess the coverage and pertinence of the core ontological level to which new concepts have been mapped and integrated. The enrichment process has demonstrated a solid foundation for hyperontology, particularly in the core and upper layers, which remained intact, and the efficient reuse of semantic relations.

**Table 11.** Some metrics for new concepts and relationships presented per cancer type.

New cancer type or subtype	Terms		New concepts (semantic mapping−direct), n	Superclasses		Ancestors		Relations	
	Total (metadata+value), n	New, n (%)		Direct, n	Reused, n (%)	All, n	Reused, n (%)	New, n	Reused, n
Glioblastoma (brain cancer)	124	109 (88)	128	149	64 (40)	1133	618 (55)	0	6
Thyroid cancer	36	15 (42)	15	20	14 (70)	173	127 (73)	0	5

## Discussion

### Related Work

Various ontologies have been developed to cover the oncology and medical imaging domains, such as the O3 [18,19], the ROO [20], and the ROS ontology [21]. O3 [18,19] is an expert-centered ontology developed using a top-down iterative approach that relies on a development process involving intervention and expertise from medical experts and stakeholders. It covers prostate, breast, and head and neck cancers, defining common aspects of oncology, including imaging, pathology, medical oncology, surgery, and radiation therapy, and integrating with standards such as SNOMED-CT, NCIT, and mCODE. ROO [20], designed with the Protégé editor, provides basic concepts, relationships, and properties for radiation oncology. To develop the ontology, data have been collected from various datasets, and a high-level structure has been established based on the Semantic Types (classes) and Relations (properties) as defined in the UMLS. The ROO ontology considered concept reuse from NCIT, ICD-10, and SEDI [33]. ROS [21], developed using Protégé, combines a bottom-up approach to identify and cluster terms into concepts, with a top-down process to organize these concepts and design the ontology. Classes are linked to the UMLS and SNOMED-CT. Several cancer types are covered in ROS, including those of the head, neck, thorax, abdomen, pelvis, limbs, and spine. However, these types are not explicitly represented in the ontology model. Alternatively, the anatomical structures associated with these cancer types have been specified. In contrast to ROO, which defines some imaging procedures mapped to NCIT (eg, Computed Tomography and Magnetic Resonance Imaging), O3 implicitly represents imaging information by associating it with diagnostic and staging information through annotations, and ROS lacks representation of the imaging context.

For specific cancer types, several ontologies have been proposed, including Prostate Cancer Ontology for prostate cancer [66], Head and Neck Cancer Ontology for head and neck cancer [67], Reference Data Model for colorectal cancer [68], Lung Cancer Audit Database for lung cancer [69], Liver Case Ontology for liver cancer [70], Thyroid Cancer Ontology for thyroid cancer [71], and the Cancer Cell Ontology for Leukemia [72]. Additionally, ontology-based approaches have been designed to support specific aspects in oncology, such as diagnosis and treatment [73] (liver cancer), TNM staging [74,75], cancer screening [76] (breast cancer), and cancer image annotation [77] (breast cancer).

A common drawback of these works is the lack of a clear, systematic approach to outlining the various steps of the ontology development process. Particularly, ontological conceptual modeling is neglected, despite its fundamental importance for developing ontologies that facilitate semantic interoperability in health care domains [78]. This gap arises from the complexity of incorporating high-level theoretical concepts grounded in foundational ontologies. However, neglecting this approach can lead to an inaccurate semantic representation of concepts. For instance, in ROO [20], diseases, including neoplasms, are classified under “Event” as “Phenomenon or Process” (independent entities), which contradicts the foundational specification of disorders or diseases as dispositions (nonindependent entities inherent in the individual), such as in the Basic Formal Ontology [79] and the UFO [39]. Besides, mappings to terminologies or vocabularies have been established in these ontologies without considering the heterogeneity of semantic interpretations of different concept categories. Finally, while some ontologies consider expert supervision [18,19,66,67], others [21,69,70,73] have neglected the clinical expertise and validation required throughout the ontology-building process, especially in the medical domain [80]. Consequently, reusing or generalizing the proposed approaches limits the faithfulness and reusability of the resulting ontologies.

What differentiates our method is the provision of a systematic, well-founded, and detailed approach. A step-by-step ontology development process is provided, composed of (1) a solid foundation and grounding in oncology and medical imaging using high-level conceptual modeling language, (2) semantic mappings across multiple standards, and (3) ontology evaluation and validation under the supervision of domain experts. Moreover, the ontology model encompasses various cancer types and their associated aspects and relationships, with a precise level of detail, while semantically harmonizing their heterogeneous interpretations. Finally, the hyperontology is richly axiomatized, including restrictions that improve its formal semantics, support automated reasoning, and enhance query precision. Table 12 summarizes the main differences among the compared ontologies (publicly available on the National Center for Biomedical Ontology BioPortal [81]), focusing on specific criteria: cancer types, imaging coverage, approach, maximum depth, axiomatization (equivalence, disjointness, and restriction axioms), mappings with standards, annotations, and concept overlap with the EUCAIM hyperontology (intersection of concepts). The shared concepts are either generic domain entities (eg, patient, anatomical structure, lesion, biopsy, chemotherapy, laterality, and computed tomography) or values (eg, male, female, high, absent, and bilateral).

**Table 12.** A comparison of related works focusing on specific criteria.

Ontology	Cancer types	Imaging	Approach	Depth	Axioms (n)	Mapping	Annotation	Concept overlap
O3^a^ [18,19]	Prostate Breast Head and neck	Annotations	Top-down Medical experts Stakeholders	2	Equivalence (2)	SNOMED^b^, NCIT^c^, mCODE^d^	8943	n=2 (patient, image)
ROO^e^ [20]	Lung Rectum	Radiology (procedures)	Use-case-driven development process	11	Disjointness (4) Equivalence (19)	UMLS^f^, NCIT, ICD-10^g^, SEDI^h^, ROO	4718	n=94 (eg, anatomical structure, anterior resection of rectum, biopsy, chemotherapy, and laterality)
ROS^i^ [21]	Abdomen Head Neck Spine Skin	N/A^j^	Top-down Bottom-up	8	N/A	UMLS, SNOMED	886	n=0
PCAO^k^ [66]	Prostate	Radiology (procedures)	Bottom-up	8	Restrictions (40)	NCIT	4713	n=23 (eg, bilateral, central zone, needle biopsy of prostate, and radical prostatectomy)
HeNeCOn^l^ [67]	Head and neck	N/A	Bottom-up	5	Equivalence (25)	ICD-10	2950	n=52 (eg, 3, absent, alive, bilateral, biopsy, female, and high)
TCO^m^ [80]	Thyroid	Radiology (procedures)	Bottom-up	8	Equivalence (571)	NCIT	3966	n=31 (eg, age, benign, resection, chemotherapy)
LiCO^n^ [70]	Liver	N/A	Data collected from real patients	3	Disjointness (50) Equivalence (27)	RadLex^o^, SNOMED, LOINC^p^	169	n=5 (cystic, lesion, liver, polyp, solid)
Hyperontology [40]	22 cancer types (eg, prostate, breast, colon, rectum, liver, pancreas, lung, bone, cervix, ovary, brain)	Radiology Digital pathology	Hybrid (Top-down+bottom-up) Modularization Layering Ontology-driven conceptual modeling Semantic patterns Medical expert supervision	24	Disjointness (11) Equivalence (119) Restrictions (31,369)	OMOP^q^, FHIR^r^, DICOM^s^, mCODE, SNOMED, ICD-10, ICD-O-3^t^, LOINC, NCIT, RxNorm, NAACCR^u^, UCUM^v^, RadLex, DCM^w^	41,555	N/A

a O3: Operational Ontology for Oncology.

b SNOMED: Systematized Nomenclature of Medicine.

c NCIT: National Cancer Institute Thesaurus.

d mCODE: Minimal Common Oncology Data Elements.

e ROO: Radiation Oncology Ontology.

f UMLS: Unified Medical Language System.

g ICD-10: International Classification of Diseases, 10th Revision.

h SEDI: Semantic DICOM Ontology.

i ROS: Radiation Oncology Structures.

j N/A: not applicable.

k PCAO: Prostate Cancer Ontology.

l HeNeCOn: Head and Neck Cancer Ontology.

m TCO: Thyroid Cancer Ontology.

n LiCO: Liver Case Ontology.

o RadLex: radiological lexicon.

p LOINC: Logical Observation Identifiers Names and Codes.

q OMOP: Observational Medical Outcomes Partnership.

r FHIR: Fast Healthcare Interoperability Resources.

s DICOM: Digital Imaging and Communications in Medicine.

t ICD-O-3: International Classification of Diseases for Oncology, 3rd Edition.

u NAACCR: North American Association of Central Cancer Registries.

v UCUM: Unified Code for Units of Measure.

w DCM: DICOM controlled terminology.

### Contributions

This study has two primary contributions: (1) a novel methodology for developing and evaluating well-founded biomedical ontologies applied to oncology and medical imaging and (2) a FAIR-compliant hyperontology that efficiently covers these domains and also facilitates semantic interoperability by integrating and harmonizing different types of real-world clinical, biological, and imaging data derived from disparate and heterogeneous cancer image data models.

The proposed methodology provides a detailed, systematic approach to address domain heterogeneity and complexity. It combines SABiO [10] and Neon [37] methodologies in a hybrid, requirement-driven, and conceptually and formally well-founded methodology. While the ontological requirements are expressed through CQs in the ORSD document, the ontology is structured into distinct layers and generic modules to semantically separate its content. This strategy helped simplify the ontology development process, address domain complexity, and ensure the ontological content’s reusability and extensibility. The top-down process that grounds the ontology in oncology, using the high-level conceptual modeling language OntoUML, faithfully reflects the real application domains, including concepts and relationships, and supports extending and enriching the ontology without altering its core content. The bottom-up process supports the development of the ontology taxonomy, drawing on knowledge from AI4HI and its associated mappings to health care and imaging standards. Additionally, the approach offers multiple methods for evaluating and validating the hyperontology in collaboration with domain experts. We also demonstrated the hyperontology extension to new cancer types without altering the core content, which makes our approach generalizable and our ontology efficiently reusable.

By applying this methodology, we obtained a FAIR-compliant hyperontology that accurately captures and represents key concepts and relationships across oncology and medical imaging. The resulting ontology encompasses various cancer types, each with its distinct heterogeneous interpretations. The Ontology Web Language ontology file, the ORSD, and the complete documentation are available at Zenodo [40] (the “F” and “A” of FAIR*—Findable* and *Accessible*). The hyperontology is also enriched with logical definitions, which enable the semantic harmonization and interoperability of heterogeneous data, as well as the understanding and standardization of ambiguous or incomplete data. Furthermore, the ontology model establishes syntactic and semantic mappings across standard terminologies and health care and imaging standards (OMOP, FHIR, and DICOM), ensuring effective data sharing and integration (the “I” of FAIR*—Interoperable*). The EUCAIM hyperontology serves as both a domain knowledge base and an application-oriented model, supporting various EUCAIM components, including federated query, image annotation or segmentation, and the ETL process (the “R” of FAIR*—Reusable*). Finally, populating the hyperontology with real-world cancer scenarios and extending it to new cancer types has also demonstrated its reusability and extensibility.

### Limitations

Despite its contributions, the hyperontology experiences several limitations, primarily related to the rapid evolution of medical and imaging knowledge in oncology and medical imaging, which makes its sustainability and scalability challenging. For instance, new cancer molecular subclassifications, biomarkers, treatments (eg, cancer vaccines), imaging procedures or techniques (eg, molecular imaging and fusion techniques), and other related developments are continuously emerging. This necessitates ongoing updates to the ontology content, especially at the domain-specific level. In terms of semantic context, various patterns or logical definitions are required to harmonize or standardize data, which can lead to excessive growth in the ontology’s complexity and size. Additionally, the semantic classification of concepts is continually evolving, whether in standard terminologies, such as ICD-10, *International Classification of Diseases for Oncology* (ICD-O), and SNOMED-CT, or in health care data models, such as OMOP. For instance, at the time of data collection, ICD-10 and ICD-O (currently in version 3) were used to represent or map cancer types and pathology codes, respectively. However, periodic updates have been performed, and new versions have emerged, such as *International Classification of Diseases, 11th Revision* (ICD-11) (introduced in 2019) and *International Classification of Diseases for Oncology, 4th Edition* (ICD-O-4; in progress), which expand the scope and classifications of health conditions and pathology, posing a challenge for data harmonization, as some categories or entities are represented differently across the various versions.

Additionally, inconsistent classifications of concepts may arise in terminologies or data models, which can impact semantic coherence and interoperability. For instance, in OMOP, secondary or metastatic neoplasms, such as “*Metastatic malignant neoplasm to liver*” (SNOMED-CT, 94381002), were classified as primary neoplasms, such as “*Malignant neoplasm of liver*” (SNOMED-CT, 93870000). The opposite is also detected, such as the case of “*Primary malignant neoplasm of breast with axillary lymph node invasion*” (SNOMED, 1082901000112103), which was previously classified as a subclass of “*Metastatic malignant neoplasm to axillary lymph nodes*” (SNOMED-CT, 94181007). These classifications, adopted in previous versions of the hyperontology, are semantically incoherent, leading to inconsistencies and logical contradictions, particularly regarding the disjointness between primary and secondary conditions. Further, the issue is resolved in OMOP. Accordingly, the specification of these concepts has been adjusted in the hyperontology. However, tracking these upgrades is time-consuming and challenging, requiring continuous effort and intervention from domain experts.

In DICOM, nonstandardized terms or values are used to represent different imaging aspects. Representing these elements in the hyperontology requires specific effort, with expert assistance, to accurately incorporate them into the semantic context and to associate them with various ontological aspects and standards. For instance, “Digitized hematoxylin-eosin (HE) slides” is a value commonly associated with the DICOM imaging modality for digital pathology, especially in the newly provided datasets. However, semantic clarification is necessary to accurately classify the term and to integrate the related concept into the imaging module’s taxonomy at the appropriate level of detail. In what follows, we provide a logical definition, expressed in Description Logic, clarifying the semantic meaning of *Digitized hematoxylin-eosin (HE) slides* in terms of standard concepts:

- *Digitized hematoxylin-eosin (HE) slides* ≡ *“Whole Slide Imaging*” ⊓ ∃*involve_staining_method*.”*Hematoxylin and eosin stain method*”

Semantically, *Digitized hematoxylin-eosin (HE) slides* is a *Whole Slide Imaging* (DCM, 112703) technique, involving the *Hematoxylin and eosin stain method* (SNOMED-CT, 104210008) as a staining method. By applying this pattern, the concept is classified under *Whole Slide Imaging*, a type of *Digital Microscopy* (NCIT, C190537)*,* enriching the semantic content and enhancing the precision of terms. Nevertheless, integrating semantic patterns for each (ambiguous) value or term will increase the ontology’s semantic and logical complexity, making it difficult to handle, especially for nonexpert users and lightweight applications.

Although the validation strategy used in this work combines automated reasoning, expert-driven evaluation, and CQ testing, several limitations should be acknowledged. First, expert-based validation may introduce interpretative bias. Domain experts necessarily rely on their own conceptualizations of biomedical entities and relationships, which may differ across institutions, specialties, or research traditions. While the involvement of multiple experts mitigates this risk, the ontology should be considered a consensus representation rather than a definitive model of oncological knowledge. Second, reasoning-based verification using the HermiT reasoner ensures logical consistency and class satisfiability. However, scalability challenges may emerge as the ontology grows or is integrated with large external ontologies or instance data. Future work will explore alternative reasoning strategies to ensure computational scalability within large-scale infrastructures such as EUCAIM. Third, the current validation process focuses on functional adequacy and logical consistency rather than formal comparative benchmarking against ontology evaluation frameworks. While CQs and expert review provide strong evidence of domain adequacy, systematic comparative evaluation with established ontology assessment tools represents an important direction for future work. Finally, the ontology reflects the current scope of EUCAIM data integration requirements. As oncology data standards evolve, iterative refinement and community-driven validation will be essential to maintain semantic alignment and interoperability.

### Conclusions and Future Work

In the health care domain, semantic interoperability—a core principle of FAIR—is gaining significant attention for its essential role in ensuring the seamless, effective integration of information systems. However, this principle is partially achieved through the use of terminologies and common data standards that define the syntactic and semantic structure of data. Semantic aspects are interpreted and represented differently and heterogeneously across standards, creating conflicts and ambiguities in data interpretation and standardization. Ontologies are a prominent solution for bridging semantic gaps, clarifying the exact meaning of data, and harmonizing exchange among disparate data sources.

The EUCAIM hyperontology is a valuable effort that provides a unifying framework to enhance semantic interoperability and facilitate communication among disparate and heterogeneous cancer image data models. To the best of our knowledge, the hyperontology is the first FAIR-compliant biomedical ontology that explicitly represents the essentials of oncology and medical imaging with a precise level of detail. It also encompasses multiple cancer types, accurately reflects domain specificity and complexity, and ensures mappings to health care and imaging standards. Developed from real-world clinical, biological, and imaging data and grounded in well-founded semantic and conceptual methods, the hyperontology faithfully captures the reality of the application domain. The ontology model and documentation are publicly available and accessible with persistent identifiers [40]. In addition, a structured approach is deployed to manage the hyperontology sustainability, versioning, and governance. Sustainability includes comprehensive documentation of the ontology scope and description, concept definitions, and use examples, as well as a maintenance strategy supported by domain experts. Versioning persists to track changes, such as new cancer types or imaging modalities provided by new data holders. Governance will be maintained through an operational entity for EUCAIM, including ontology and domain experts, to ensure a controlled ontology evolution.

Future work will focus on maintaining the oncology dynamic aspects by ontologically analyzing, at the generic level, disease and treatment episodes from the OMOP CDM oncology extension [36,82]. This strategy, which complements the mCODE-based context discussed in this study, will align with the data-driven prediction of treatment efficiency and cancer survival, the outcome, which depends on the quality of health care data consistently collected and organized across the patient’s trajectory through the disease into episodes of diagnosis, treatment, and outcome with disease-free survival, relapse or recurrence, and progression [82]. The hyperontology is being integrated in various EUCAIM applications, including the ETL process, federated query, and image annotation or segmentation. This will help assess the applicability and performance of hyperontology in real-world tasks. In this context, future work will also focus on the evaluation of ontology functional dimension [83] using quantitative metrics, such as precision and recall [84]. Establishing such a metric, which can be measured against experts’ judgments [83], requires developing a reference dataset and expert-validated annotations. This will enable a more comprehensive assessment of ontology coverage and accuracy. Furthermore, advanced validation is necessary, notably to support federated processing and decision-support systems.

## Abbreviations

AI4HI: AI for Health Imaging
BI-RADS: Breast Imaging Reporting and Data System
BRCA2: breast cancer gene 2
CDM: Common Data Model
CQ: competency question
CT: computed tomography
DICOM: Digital Imaging and Communications in Medicine
DSL: domain-specific layer
ER: estrogen receptor
ETL: extract, transform, and load
EUCAIM: Cancer Image Europe
FAIR: Findable, Accessible, Interoperable, Reusable
FHIR: Fast Healthcare Interoperability Resources
HE: hematoxylin-eosin
HER2: human epidermal growth factor receptor 2
HR: hormone receptor
ICD-10: International Classification of Diseases, 10th Revision
ICD-11: International Classification of Diseases, 11th Revision
ICD-O: International Classification of Diseases for Oncology
ICD-O-3: International Classification of Diseases for Oncology, 3rd Edition
ICD-O-4: International Classification of Diseases for Oncology, 4th Edition
LI-RADS: Liver Imaging Reporting and Data System
LI-RADS: Liver Imaging Reporting and Data System
LOINC: Logical Observation Identifiers Names and Codes
mCODE: Minimal Common Oncology Data Elements
MR: magnetic resonance
MRI: magnetic resonance imaging
NCIT: National Cancer Institute Thesaurus
O3: Operational Ontology for Oncology
OMOP: Observational Medical Outcomes Partnership
ORSD: Ontology Requirements Specification Document
OWL2: Web Ontology Language 2
PET: positron emission tomography
PI-RADS: Prostate Imaging Reporting and Data System
PR: progesterone receptor
PSA: prostate-specific antigen
RadLex: radiological lexicon
ROO: Radiation Oncology Ontology
ROS: Radiation Oncology Structures
SABiO: Systematic Approach for Building Ontologies
SEDI: Semantic DICOM Ontology
SKOS: Simple Knowledge Organization System
SNOMED: Systematized Nomenclature of Medicine
SNOMED-CT: Systematized Nomenclature of Medicine Clinical Terms
SPARQL: SPARQL Protocol and Resource Description Framework Query Language
TP53: tumor protein p53
UFO: Unified Foundational Ontology
UMLS: Unified Medical Language System
WSI: Whole Slide Imaging
